# The ambiguous ripening nature of the fig (*Ficus carica* L.) fruit: a gene-expression study of potential ripening regulators and ethylene-related genes

**DOI:** 10.1093/jxb/erv140

**Published:** 2015-05-08

**Authors:** Zohar E. Freiman, Yogev Rosianskey, Rajeswari Dasmohapatra, Itzhak Kamara, Moshe A. Flaishman

**Affiliations:** ^1^Institute of Plant Sciences, Agricultural Research Organization, P.O. Box 6, Bet-Dagan 50250, Israel; ^2^The Robert H. Smith Institute of Plant Sciences and Genetics in Agriculture, Faculty of Agriculture, Food and Environment, The Hebrew University of Jerusalem, P.O. Box 12, Rehovot 76100, Israel

**Keywords:** 1-Methylcyclopropene (1-MCP), ethylene, *Ficus carica*, fruit ripening, *MADS-box*, preharvest treatment.

## Abstract

The traditional definition of climacteric and non-climacteric fruits has been put into question. A significant example of this paradox is the climacteric fig fruit. Surprisingly, ripening-related ethylene production increases following pre- or postharvest 1-methylcyclopropene (1-MCP) application in an unexpected auto-inhibitory manner. In this study, ethylene production and the expression of potential ripening-regulator, ethylene-synthesis, and signal-transduction genes are characterized in figs ripening on the tree and following preharvest 1-MCP application. Fig ripening-related gene expression was similar to that in tomato and apple during ripening on the tree, but only in the fig inflorescence–drupelet section. Because the pattern in the receptacle is different for most of the genes, the fig drupelets developed inside the syconium are proposed to function as parthenocarpic true fruit, regulating ripening processes for the whole accessory fruit. Transcription of a potential ripening regulator, *FcMADS8*, increased during ripening on the tree and was inhibited following 1-MCP treatment. Expression patterns of the ethylene-synthesis genes *FcACS2*, *FcACS4*, and *FcACO3* could be related to the auto-inhibition reaction of ethylene production in 1-MCP-treated fruit. Along with *FcMADS8* suppression, gene expression analysis revealed upregulation of *FcEBF1*, and downregulation of *FcEIL3* and several *FcERF*s by 1-MCP treatment. This corresponded with the high storability of the treated fruit. One *FcERF* was overexpressed in the 1-MCP-treated fruit, and did not share the increasing pattern of most *FcERF*s in the tree-ripened fig. This demonstrates the potential of this downstream ethylene-signal-transduction component as an ethylene-synthesis regulator, responsible for the non-climacteric auto-inhibition of ethylene production in fig.

## Highlight

The ambiguous ripening nature of fig fruits contradicts the climacteric definition. One ethylene-responsive factor gene is potentially the ethylene-synthesis regulator responsible for the non-climacteric auto-inhibition of ethylene production in fig.

## Introduction

In the last few years, fruit-ripening research has challenged the classical definitions of climacteric and non-climacteric fleshy fruits (Paul *et al.*, 2012). A unique example of this controversy is the fig, *Ficus carica* L. The ripening process in fig fruit is categorized as climacteric, showing a rise in respiration rate and ethylene production at the onset of the ripening phase (Marei and Crane, 1971). Surprisingly, ripening-related ethylene production increases following pre- or postharvest 1-methylcyclopropene (1-MCP) application in an unexpected auto-inhibitory manner. Moreover, postharvest 1-MCP treatment does not affect the ripening parameters of the treated fruit (unlike other climacteric fruits), while application to fruit on the tree improves fruit-storage abilities, inhibiting deterioration with minor effects on fruit growth and ripening (Sozzi *et al.*, 2005; Owino *et al.,* 2006; Freiman *et al.,* 2012). In addition to the auto-inhibitory reaction of ethylene production in the climacteric-classified fig, other unique characteristics of this fruit differentiate it from the well-studied *Solanum lycopersicum* (tomato) climacteric model. Not only does the fig develop to its final size during ripening, the ripening process in the main summer crop is rapid, taking less than 3 days. Fruit picked before optimal maturity never reach the desirable parameters of size, colour, flavour, or texture, while fruits harvested too late tend to perish due to over-ripening and high susceptibility to pathogens (Flaishman *et al.*, 2008). By contrast, tomato, *Malus domestica* (apple), and *Musa* spp. (banana) fruits, for example, reach their final size at the mature green stage, and only then are the ripening processes initiated. Tomato fruit can be picked at the mature green stage and still develop to the red ripe stage within 10 days, whereas ripening of non-harvested tomato can take over 20 days (Yokotani et al., 2009; Van de Poel et al., 2013).

As with all *Ficus* species, the fig bears a unique closed inflorescence structure—the syconium—that is not present in any other fruit in the human diet. The multiple fig fruit is composed of small individual drupelets which develop from the ovaries enclosed in the succulent receptacle to form a single accessory fruit (Storey, 1977). Development of the fig’s female fruit is characterized by a double sigmoid growth curve comprising three phases (Marei and Crane, 1971). Phase I is characterized by a rapid growth in size; during phase II, the fruit remains nearly the same size, colour, and firmness. Phase III is the ripening phase and includes fruit growth, colour change, softening, and alteration of the pulp texture to an edible state. The parthenocarpic fruit of the purple female fig cultivar ‘Brown Turkey’ shows onset of ethylene production when the green hue of the peel starts to fade to yellow, at the transition from phase II to III. In attached ‘Brown Turkey’ figs, ethylene peaks during ripening at the commercially ripe fruit stage (50% purple peel), and declines toward the fully ripened fruit stage (100% purple peel). All ripening parameters—size, firmness, and inner texture—present differences between on-tree 1-MCP-treated and untreated fruit (Freiman *et al.,* 2012). Fig ethylene-synthesis genes—three ACC-synthase (*ACS*) genes and a single ACC-oxidase (*ACO*) gene—were isolated by Owino *et al.* (2006), and their expression patterns were studied postharvest and following several treatments. The expression patterns of *FcACS1*, *FcACS3*, and *FcACO1* were inhibited in figs following postharvest 1-MCP treatment, indicating positive regulation by ethylene (when ethylene is not sensed by the tissue, its synthesis genes are downregulated), whereas *FcACS2* expression was induced by 1-MCP, indicating negative regulation (when ethylene is not sensed by the tissue, its synthesis genes are upregulated). Recently, a transcriptome analysis of caprifig (a hermaphroditic fruit that functions as male) and fig female fruit in late phase II was published. The study identified several unigenes encoding proteins involved in ethylene synthesis and signal transduction, assembled from combined data from female ‘Houraishi’ and hermaphroditic ecotypes (Ikegami et al., 2013). A transcriptome analysis of fig (‘Brown Turkey’) during phase III development was published by Freiman *et al*., 2014. Unlike the work on ‘Houraishi’ and caprifigs, which focused on the differences between the caprifig and female fig in phase II, the transcriptome analysis of ‘Brown Turkey’ targeted ripening processes in phase III and the transition toward this phase. In the latter work, a few ethylene-synthesis genes were identified and *MADS-box* genes were isolated. The genes were analysed for their expression in the receptacle and *FcMADS* genes were classified into different *MADS-box* family clades.

The ripening cascade in the climacteric fruit model, the tomato, can be divided into three levels: transcription factors controlling the transition to ripening phase, ethylene biosynthesis and perception networks regulating ripening, and the coordinated metabolic processes of ripening (Seymour et al., 2013). Compared to ethylene synthesis and regulation, which involves *ACS*s and *ACO*s, signal transduction of ethylene is much more complex. Ethylene binds to its receptors, releasing the activation of constitutive triple response1 (CTR1), which, in the absence of ethylene, phosphorylates ethylene-insensitive2 (EIN2). In the presence of ethylene, EIN2 is not phosphorylated and its C terminus is cleaved and moves to the nucleus to induce ethylene-insensitive 3-binding F-box (EBF)1/2 degradation activity. EBF1/2 targets EIN3 and EIN3-like (EIL) for protein degradation, and release of EIN2’s C terminus by ethylene thus stabilizes EIN3/EILs, which in turn bind to ethylene-responsive factor (ERF) promoters. This is the last step in the ethylene-signal-transduction pathway: ERFs bind to downstream genes involved in metabolic ripening processes, as well as in feedback regulation of ethylene synthesis (Merchante et al., 2013).

In the present work, ethylene production and the expression of potential ripening-regulator, ethylene-synthesis, and signal-transduction genes (Supplementary Table S1) are characterized in on-tree-ripening fig fruit. In addition, the effect of preharvest 1-MCP treatment on gene expression is examined. The association between ethylene profile and specific ethylene-synthesis genes is analysed in the context of ethylene-synthesis systems. Expression of ethylene-signal-transduction elements is compared to that in other climacteric fruits with respect to feedback regulation of ethylene synthesis. The study of both ethylene-synthesis and signal-transduction pathways, in on-tree-ripening fruit and in preharvest 1-MCP-treated fruit, indicates the specific genes incorporated in fig ripening and in the auto-inhibitory response to 1-MCP application. In addition, the study pinpoints genes that are assumed to be responsible for the positive effect of preharvest 1-MCP treatment on fig-ripening parameters after storage.

## Materials and methods

### Plant material

**Fig. 1.**
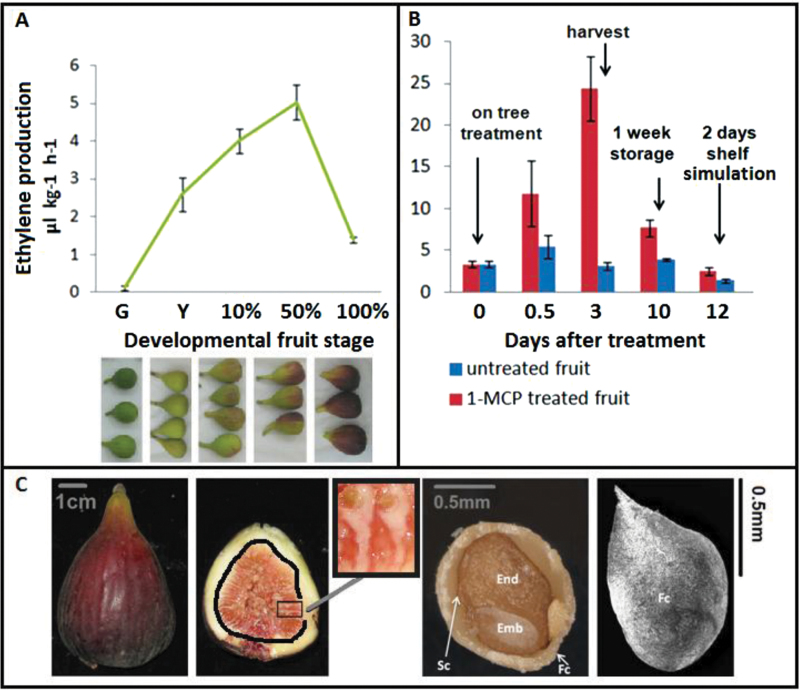
Studied fig fruit-ripening systems. (A) Ethylene production in fig fruit during on-tree ripening. Developmental fruit stages: G – green fruit, pre-ripening stage at the end of phase II; Y – yellow fruit, ripening-onset stage at the transition from phase II to phase III; 10% – 10% purple peel fruit, ripening-initiation stage; 50% – 50% purple peel fruit, commercially ripe stage; 100% – 100% purple peel fruit, fully ripe stage. Average of seven fruits per treatment/control group ± SE. (B) Ethylene production in preharvest 1-MCP-treated fruit (at ripening-onset fruit stage) followed by 1 week of cold storage and 2 days of shelf simulation Days after treatment: 0 – ripening-onset fruit before treatment; 0.5 – ripening-onset fruit after 1-MCP treatment on tree; 3 – commercially ripe fruit developed on tree; 10 – harvested commercially ripe fruit after 1 week of storage; 12 – harvested commercially ripe and stored fruit after 2 days of shelf simulation. Average of seven fruits per treatment/control group ± SE. (C) Fully ripe parthenocarpic ‘Brown Turkey’ fig syconium. Left to right – an external view of the syconium; an internal view of a longitudinal cross section of the syconium (the receptacle and inflorescence sections are separated by a thick line); a close view of the female flowers enclosed in the syconium; a stereoscope image of a longitudinal cross section of a single drupelet; a SEM image of the external of a single drupelet.

For the on-tree trial, fruit of the female fig (*Ficus carica*) cultivar ‘Brown Turkey’ were collected from a commercial orchard located near Be’er Tuvia in the southern coastal plain of Israel, 55 m above sea level. Fruits used for this study were from the summer crop, August 2013, with day temperatures of 26–32°C and night temperatures of 22–25°C. Five developmental stages were sampled for ethylene-production measurements and gene-expression quantification: green fruit – pre-ripening stage, end of phase II; yellow fruit – ripening-onset stage, transition from phase II to III; 10% purple peel – ripening-initiation stage, phase III; 50% purple peel – commercially mature stage, phase III; 100% purple peel – fully ripe stage, end of phase III (Fig. 1A).

For the preharvest 1-MCP treatment and storage trial, figs were subjected to preharvest treatment with 1-MCP in a commercial orchard located near Karmei Yosef in the Judea plain of Israel, 137 m above sea level. Fruits used for this study were from the autumn crop, November 2011, with day temperatures of 20–25°C and night temperatures of 10–15°C. Samples subjected to ethylene-production measurements and gene-expression quantification were as follows: ripening-onset fruit before treatment; fruit after overnight 1-MCP treatment/untreated tagged control; commercially mature fruit harvested 3 days after treatment/control; treated/control fruit subjected to storage; treated/control fruit subjected to shelf simulation (Fig. 1B).

### Parthenocarpic drupelet examination

For the demonstration of parthenocarpic drupelets, a longitudinal cross section of a fully ripe fig drupelet was examined under a stereoscope (Zeiss Stemi 2000-C, Zeiss, Oberkochen, Germany; Fig. 1C). An external view of the drupelet was obtained by scanning electron microscopy (SEM; Fig. 1C) as follows: parthenocarpic drupelets were fixed in Formalin-acetic acid-alcohol solution (100% acetic acid, 40% formalin, 95% ethanol at 1:2:10, v/v); dehydrated in a series of 25, 50, 75, 90 and 100% ethanol; dried in liquid CO_2_ (Bio-Rad 750 critical-point dryer, Hemel Hempstead, UK); then placed on SEM discs, coated with a 10-nm gold layer, and studied by SEM (JEOL, Tokyo, Japan) at an accelerating potential of 15kV (Kamenetsky, 1994).

### Chemicals

Required concentrations of 1-MCP were obtained from Ethyl-Block powder (Floralife, Inc., Walterboro, SC, USA), with an active ingredient content of 0.14%. The preparation was a gift from Riesel Chackvet Ltd. (Petah Tikva, Israel). The release of 1-MCP from the preparation was tested by gas chromatography as described below and found comparable to that from the commercial product Smart-Fresh (AgroFresh, Spring House, PA, USA).

### Preharvest 1-MCP treatment

Fig fruit were treated in the orchard with the 1-MCP gas as described by Freiman *et al.* (2012).

### Ethylene-production measurements

Seven fruits per stage or treatment were sampled for ethylene-production analysis, performed as described by Freiman *et al.* (2012).

### Storage trial

For the storage trial, 1-MCP was applied to the fruit after onset of chlorophyll loss, determined as a change in fruit colour from dull to light green. Fig fruit of approximately the same size (300 fruit, about 4cm in diameter) were marked on trees. Of these, 150 figs were treated with 1-MCP as described above and the other 150 were tagged as untreated controls. After the treatment, the fruit were left on the tree for an additional 3 days and then harvested and stored at the commercially mature stage, according to previously determined cultivar-specific visual and manual criteria (Rodov *et al.*, 2002), namely purple coloration on 20–70% of their surface and slight elastic yielding to mild finger pressure. For analysis, figs with coloration on 50% of their surface were arranged in cartons with plastic-cavity insert trays, each fruit in a separate cavity. The cartons, each containing 10–20 commercially mature fruit, were stored for 7 days at 1–2°C and 90–95% relative humidity, and then for 2 days at 20°C and 85% relative humidity (shelf simulation).

### RNA extraction, cDNA synthesis, and transcript isolation

For quantitative PCR analysis, total RNA was extracted according to Jaakola *et al*., 2001. RNA concentration was determined in a NanoDrop ND-1000 spectrophotometer (Wilmington, DE, USA), and its integrity was checked by running 1 µL in a 1% (w/v) agarose gel stained with bromophenol blue. Total RNA was digested with RQ-DNase (Promega, Madison, WI, USA). Complementary DNA was synthesized, using Oligo-dT primers, with the VERSO cDNA kit (Thermo Scientific, Waltham, MA, USA). The reaction was performed in a T-Gradient PCR system (Biometra, Goettingen, Germany). Newly isolated transcripts of *FcACS1L* (KP892658), *FcACS4* (KP892659), *FcACO2* (KP892660) and *FcACO3* (KP892661) were sequenced with the use of specific primers flanking the coding sequence according to the published transcriptome (Supplementary Table S2; Freiman *et al*., 2014).

### High-throughput real-time quantitative PCR

High-throughput real-time quantitative PCR was performed on a BioMark 96.96 Dynamic Array (Fluidigm Corp., San Francisco, CA, USA) with TaqMan Gene Expression Assays (Applied Biosystems, Carlsbad, CA, USA) at the Weizmann Institute of Science (Rehovot, Israel). Three biological replicates were used for each treatment and two technical replicates were analysed for each biological replicate. Primers (Supplementary Table S3) were designed with Primer3 software, and synthesized by Metabion (Steinkirchen, Germany) and Hylabs (Rehovot, Israel). Expression levels of the target genes were normalized to the control gene *actin*.

## Results

### Ethylene production of natural on-tree-ripening fig and stored fruit treated preharvest with 1-MCP

To investigate the function of ripening-regulator genes in fig fruit, two systems were examined. The first was natural on-tree fig ripening, with sampling of five fruit stages as presented in Fig. 1A. The second system examined was preharvest 1-MCP-treated fruit stored under commercial conditions (1–2°C) and subjected to shelf simulation (20°C) (Fig. 1B). As expected, in the natural on-tree-ripening fig, a rise in ethylene production was detected when fruit colour changed from green (0.11 µL kg^-1^ h^-1^) to yellow (2.6 µL kg^-1^ h^-1^). Ethylene production continued to increase toward the 50% purple stage (5 µL kg^-1^ h^-1^) and decreased at the 100% purple stage (1.4 µL kg^-1^ h^-1^). Following overnight preharvest 1-MCP treatment, ethylene production in treated yellow fruit (0.5 days after treatment) was 2.5 times higher than that in untreated fruit (Fig. 1B). This auto-inhibitory pattern following preharvest 1-MCP treatment has been previously documented, along with improved storability of the treated fruit (Freiman *et al*., 2012). In the present study, further ethylene measurements were taken: after fruit harvest, storage, and shelf simulation (Fig. 1B). High levels of ethylene were evident in the treated fruit 3 days after treatment (harvested commercially mature stage)—eight times higher than that in the untreated fruit (Fig. 1B). After 1 week of storage (10 days after treatment), ethylene levels of the treated fruit were twice as high as those of the untreated fruit and even after 2 days of shelf simulation (12 days after treatment), a difference in ethylene production was observed (1.8 times higher in treated versus untreated fruit, Fig. 1B).

### Identification of *MADS-box* and ethylene-related genes

To determine the molecular components responsible for the unique ethylene characteristics in fig and the improved storability of preharvest 1-MCP-treated fruit, a matrix of ripening-related fig genes was established. Fifty-seven fig genes homologous to *MADS-box*, ethylene-synthesis and ethylene-signal-transduction genes were subjected to gene-expression analysis (Supplementary Table S1). Gene families included *MADS-box*, *ACS*, *ETO1*-like (*EOL*), *ACO*, ethylene receptors (*ETR*, *EIN4*, and *ERS1*), *CTR*/enhanced disease resistance 1 (*EDR1*; EDR1 and CTR1 are similar and share some functions; Frye et al., 2001), *EIN2*, *EBF1*, *EIL*, and the *ERF* family. Eight of the *FcMADS-box* genes have been isolated previously as potential regulators of ripening and ethylene production. Six of them were further examined here for their expression levels: *FcMADS1* from the AGL6 subfamily, *FcMADS2* and *FcMADS3* from the SQUAMOSA subfamily, *FcMADS4* from the STMADS11 subfamily, and *FcMADS6* and *FcMADS8* from the SEP subfamily (Freiman et al., 2014).

To explore ethylene synthesis, the expressions of four *FcACS* family members were analysed: *FcACS1L*, *FcACS2*, *FcACS3*, and *FcACS4*. Partial transcript sequences of *FcACS1, FcACS2*, and *FcACS3* were previously published by Owino *et al.* (2006). A longer transcript of *FcACS1* (*FcACS1L*, Supplementary Fig. S1) showed 99% identity to the published sequence, with a single nucleotide addition resulting in a frame shift to complete the full-length transcript. *FcACS4* is newly presented here. Phylogenetic analysis revealed that *FcACS1L* and *FcACS2* are type 1 *ACS*s, whereas *FcACS3* and *FcACS4* are type 2 (Supplementary Fig. S2). Homologues of *ETO1* and *EOL,* which target type 2 ACSs for degradation via the proteasome (McClellan and Chang, 2008), were identified in the developing fig transcriptome: *FcEOL1* and *FcEOL2*. The last group of ethylene-synthesis genes isolated was from the *ACO* family: *FcACOL*, *FcACO2*, and *FcACO3*. The isolated *FcACOL* was 96% identical to the *FcACO1* sequence previously published by Owino *et al.* (2006) and 100% identical to the full-length *FcACOL* transcript sequence submitted to the National Center for Biotechnology Information (NCBI) with no additional information (mRNA GenBank accession no. AB307720.1 2007, Supplementary Fig. S3), while *FcACO2* and *FcACO3* are newly presented here.

To study ethylene-signal-transduction elements in fig fruit, genes from this pathway were identified in the developing fig transcriptome. Upstream components, acting as ethylene-response inhibitors, were identified, including four receptors (*FcETR1*, *FcETR2*, *FcEIN4*, and *FcERS1*) and four *CTR1* genes (*FcCTR1*, *FcCTR2*, *FcCTR3*, and *FcEDR1*). The positive signal-transduction regulator *FcEIN2* was identified as a single gene, as found in *Arabidopsis* and tomato (Gapper *et al.*, 2013). Downstream of *EIN2*, the ethylene-response inhibitor *FcEBF1* was also identified. Three possible targets of *FcEBF1*, namely *EIN3*/*EIL*, were detected: *FcEIL1*, *FcEIL2*, and *FcEIL3*. These components are targeted for degradation by *EBF1* and are positive regulators of the ethylene response via activation of a large group of *ERF*s. Twenty-seven *FcERF*s were traced in the developing fig transcriptome, and their transcript quantification completes the set of ethylene-signal-transduction genes in fig fruit.

### Expression of *MADS-box* and ethylene-synthesis-related genes in the fig inflorescence and receptacle during natural on-tree ripening

**Fig. 2.**
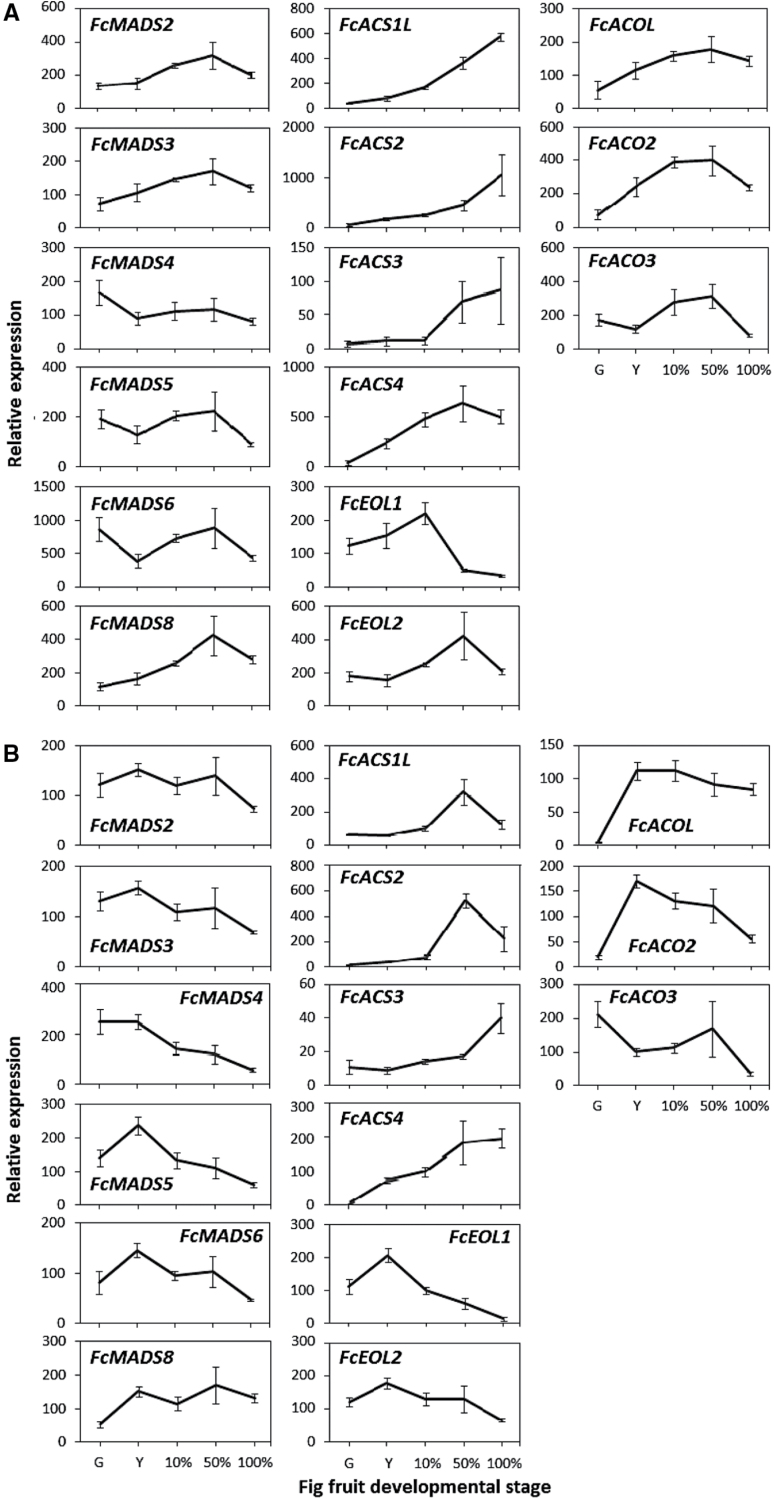
Expression patterns of *FcMADS-box* and ethylene-synthesis-related genes (*ACS*s, *EOL*s, and *ACO*s) in on-tree-ripening fig fruit. (A) Gene expression in the inflorescence. (B) Gene expression in the receptacle. Developmental fruit stages are as described in Fig. 1A. Average ± SE of three fruits per stage, each in two technical replicates.

To find potential ripening-regulator genes in fig fruit, the expression of *FcMADS* and ethylene-synthesis-related genes was examined during natural on-tree ripening (Fig. 1A). For gene-expression analysis in natural ripening fig, the fruit inflorescence and receptacle were separated. Inflorescence tissue included non-pollinated drupelets, while the receptacle tissue included the coloured outer peel of the fruit (Fig. 1C). The exterior and interior of a single parthenocarpic drupelet can be seen in Fig. 1C. As shown in Fig. 2A, in the fig inflorescence, three *MADS-box* genes—*FcMADS2*, *FcMADS3*, and *FcMADS8*—were upregulated from the yellow stage toward the 50% purple stage, followed by a minor decrease in expression at the 100% purple stage. *FcMADS8* showed a 3.5-fold change in expression from the green stage to the 50% purple stage, while both *FcMADS2* and *FcMADS8* exhibited higher expression levels than *FcMADS3*. Three genes (*FcMADS4*, *FcMADS5*, and *FcMADS6*) were downregulated from the green stage to the yellow stage, while *FcMADS5* and *FcMADS6* presented minor increases at the 10% purple stage and a decrease at the 100% purple stage. Constituting the first exclusive ethylene-synthesis step, all *FcACS* transcripts in the inflorescence showed low expression levels at the green stage, which were enhanced at some point during fig ripening. *FcACS4* gene expression showed the first prominent rise at the yellow stage (fold change of 16.4), and continued to rise at the 10% stage. Both *FcACS1L* and *FcACS2* exhibited their major changes at the latter stages of the ripening process (fold changes of 3.4 and 4, respectively). Expression levels of *FcACS3* were lower than those of the other *ACS* genes; nevertheless, *FcACS3* transcription was upregulated 5.7-fold at the 50% purple stage. Post-translational regulators of type 2 ACS proteins—*FcEOL1* and *FcEOL2*—showed transcription peaks at the 10% and 50% purple stages, respectively. Like the *FcACS*s, *FcACO*s presented increasing transcription patterns as the fig ripened. Expression of both *FcACOL* and *FcACO2* was enhanced from the green stage to the 50% purple stage (3.2- and 5.3-fold, respectively), while minor decreases were observed at the 100% purple stage. *FcACO3* transcription displayed a different pattern, with a minor decrease at the yellow stage followed by upregulation at the 10% purple stage (fold change of 2.5). The high transcript level of *FcACO3* was still observed at the 50% purple stage but decreased 3.7-fold at the 100% purple stage.

In the fig receptacle (Fig. 2B), three *MADS-box* genes—*FcMADS2*, *FcMADS3*, and *FcMADS4*—were downregulated from the yellow stage toward the 100% purple stage. *FcMADS5* and *FcMADS6* showed similar patterns from the yellow stage on, but the decreases in transcript levels followed increases from the green to yellow stage for both genes. The expression of *FcMADS8* remained high after rising at the yellow stage (fold change of 2.8). Though the rate of *FcMADS8* upregulation in the receptacle resembled that in the inflorescence, transcript levels in the inflorescence were higher (Fig. 2A). Transcription of *FcACS* genes in the receptacle shared a common pattern with the inflorescence, i.e. low expression levels at the green stage and enhancement at some point during fig ripening. Here, too, *FcACS4* expression showed the first prominent rise at the yellow stage (fold change of 77), but continued to rise toward the 100% purple stage with 215 times higher levels than at the green stage. Both *FcACS1L* and *FcACS2* exhibited enhanced expression at the 50% purple stage (fold changes of 3.3 and 6.3, respectively) followed by a decrease at the 100% purple stage (fold changes of 2.5 and 2.3, respectively). Expression levels of *FcACS3* rose at the 100% purple stage, albeit to lower levels than the other *ACS*s. In general, transcription levels of *ACS*s in the receptacle were lower than in the inflorescence. The post-translational regulators of type 2 ACS proteins—*FcEOL1* and *FcEOL2*—both showed peak transcription at the 10% purple stage. Two *FcACO*s—*FcACOL* and *FcACO2*—presented low expression at the green stage and enhanced transcription levels at the yellow stage (fold changes of 22 and 9, respectively). *FcACOL* expression remained high, while that of *FcACO2* decreased toward the 100% purple stage. *FcACO3* expression displayed a different pattern, with downregulation at the yellow stage and again at the 100% purple stage.

### Expression of ethylene-signal-transduction genes in the fig inflorescence and receptacle during natural on-tree ripening

**Fig. 3.**
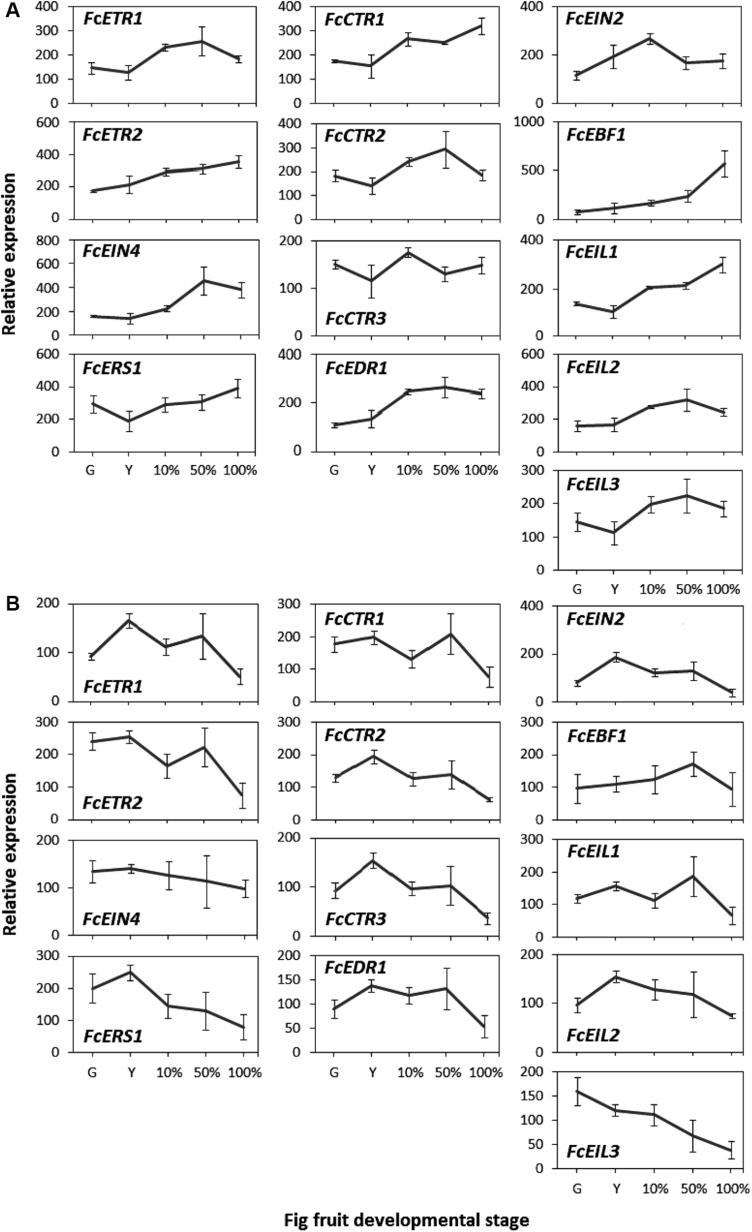
Expression patterns of ethylene-signal-transduction-related genes in on-tree-ripening fig fruit. Ethylene-receptor genes (*FcETR1*, *FcETR2*, *FcEIN4*, and *FcERS1*), *CTR1-like* genes (*FcEDR1* and *FcCTR1–3*), *FcEIN2*, *FcEBF1*, and *EIN3/EIL* (*FcEIL1–3*). (A) Gene expression in the inflorescence. (B) Gene expression in the receptacle. Developmental fruit stages are as described in Fig. 1A.

To determine the function of the ethylene-signal-transduction pathway during fig ripening, expression of its genes was examined (Fig. 3). In the inflorescence (Fig. 3A), ethylene receptors showed moderate increases in expression, with *FcEIN4* presenting the largest change (3.3-fold) from the yellow stage to the 50% purple stage. The *CTR*s—*FcCTR1*, *FcCTR2*, and *FcEDR1*—also showed increased expression in the inflorescence during fig ripening. *FcCTR1* exhibited the highest change (6-fold) from the yellow stage to the 100% purple stage, and *FcCTR3* expression showed minor changes. *FcEIN2* was upregulated from the green stage to the 10% stage (2.3-fold) and moderately downregulated at the 50% stage (1.6-fold). Transcription of *FcEBF1* gradually increased with fig ripening, with the transcript level at the 100% purple stage being 7.5 times higher than at the green stage. *FcEIL* genes were also upregulated in the inflorescence during ripening, with *FcEIL3* exhibiting the highest change (2.7-fold) from the yellow stage to the 50% purple stage.

In the receptacle, the expression patterns of all ethylene-signal-transduction genes differed from their inflorescence profiles (Fig. 3B). With the exception of *FcETR1*, there was no detectable increase in expression of the ethylene receptors during ripening. A minor (1.7-fold) rise in *FcETR1* expression in the receptacle was restricted to the yellow stage. Later on, the *FcETR1* transcript level decreased: at the 100% purple stage, it was even lower than its starting point—the green stage. Both *FcETR2* and *FcERS1* shared this decrease from the yellow stage to the 100% purple stage (fold changes of 3.4 and 3.1, respectively). No apparent change was observed in *FcEIN4* expression in the receptacle. The *CTR* genes—*FcCTR2*, *FcCTR3*, and *FcEDR1*—also showed gradual decreases in expression from the yellow stage to the 100% purple stage (fold changes of 3, 4, and 2.5, respectively). *FcCTR1* showed a 2.7-fold decline in transcription from the 50% purple stage to the 100% purple stage. *FcEIN2* was upregulated 2.3-fold from the green stage to the yellow stage and then gradually downregulated toward the 100% purple stage (4.7-fold). Transcription levels of *FcEBF1* presented minor changes in the receptacle as the fig ripened, while *FcEIL*s exhibited different transcription patterns. *FcEIL1* expression was downregulated 2.7-fold at the 100% purple stage, *FcEIL2* showed a minor expression peak at the yellow stage, and *FcEIL3* transcription was 4 times lower at the 100% purple versus green stage.

**Fig. 4.**
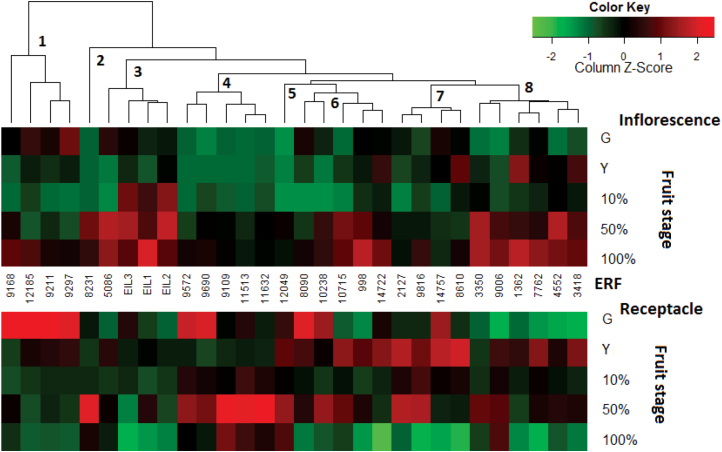
Expression patterns of ethylene-responsive factor genes (*ERF*s) in on-tree-ripening fig fruit. Numbers indicate clusters. Developmental fruit stages are as described in Fig. 1A.

Comprising the last step in ethylene-signal transduction, the transcription patterns of the 27 examined *FcERF*s differed between the inflorescence and the receptacle (Fig. 4). The *FcERF*s and *FcEIL*s were clustered according to their expression patterns. Cluster 1 consisted of *FcERF*s with higher expression levels in the receptacle than in the inflorescence at the green stage, but higher levels in the inflorescence at the 100% purple stage. Cluster 2 only contained *FcERF8231*, which showed similar patterns in both sampled tissues. Cluster 3 (which included the three *FcEIL*s) and cluster 8 showed higher transcription levels in the inflorescence at later ripening stages, whereas cluster 4 exhibited the opposite trend, with higher expression levels in the receptacle at the later ripening stages. Cluster 5 only contained *FcERF12049*, which showed higher transcription levels in the receptacle during all ripening stages. Cluster 6 contained genes with elevated transcript levels toward ripening completion in the inflorescence compared to the receptacle, in which levels were high at the yellow and 50% purple stages. Minor changes in transcription in the inflorescence were evident for the genes in cluster 7, while in the receptacle, elevated transcription was evident at the yellow stage and at the 50% purple stage in the case of *FcERF2127* and *FcERF9816*. To associate *FcEIL*s and their targets, *FcERF*s, *FcEIL*s were also clustered with *FcERF*s. As mentioned, all three *EIL*s were designated to cluster 3.

### Gene expression of *MADS-box* and ethylene-synthesis-related genes following preharvest 1-MCP treatment, storage, and shelf simulation

**Fig. 5.**
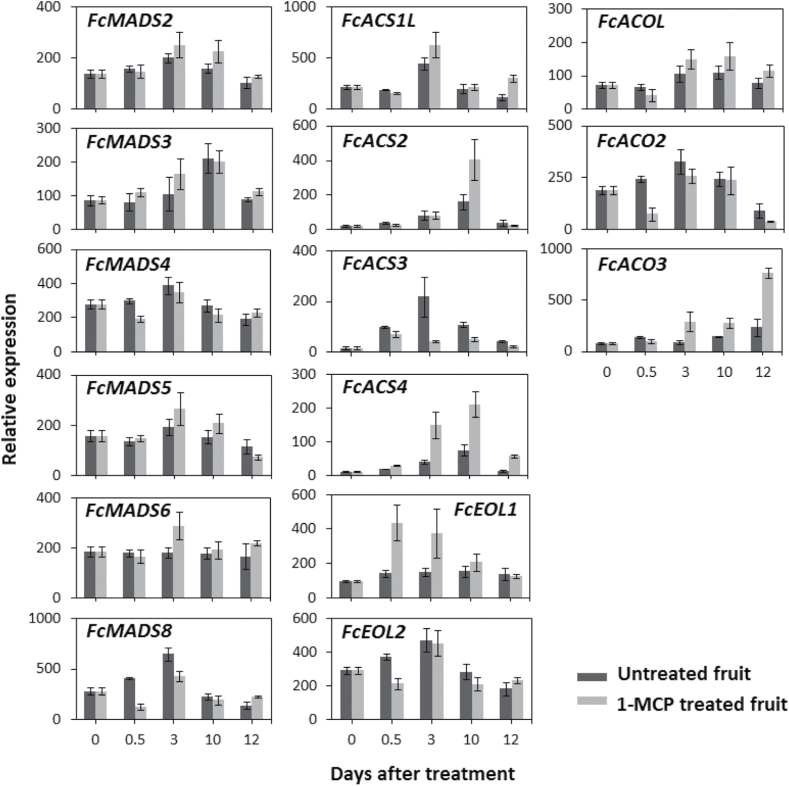
Expression patterns of *FcMADS-box* genes and ethylene-synthesis-related genes (*ACS*s, *EOL*s, and *ACO*s) in preharvest 1-MCP-treated and untreated fruit (at ripening-onset fruit stage) followed by 1 week in cold storage and 2 days of shelf simulation. Days after treatment are as described in Fig. 1B. Average ± SE of three fruits per treatment/control, each in two technical replicates.

To determine ethylene sensitivity of ripening-regulation pathways in fig and their relationship with the improved storability of preharvest 1-MCP-treated fruit, the expression of *FcMADS* and ethylene-synthesis-related genes was examined. Gene-expression analysis in preharvest 1-MCP-treated stored fruit was performed on whole fruit; sampling was performed as presented in Fig. 1B. As shown in Fig. 5, out of the six *MADS-box* genes examined, only *FcMADS3* was upregulated after storage in untreated as well as treated fruit. Expression of *FcMADS2*, *FcMADS4*, *FcMADS5*, and *FcMADS8* declined after storage and even further after shelf simulation in untreated fruit, whereas the *FcMAD6* expression level did not change. The effect of 1-MCP treatment was observed in the transcription patterns of *FcMADS4*, *FcMADS6*, and *FcMADS8*. While *FcMADS4* and *FcMADS8* expression was downregulated 0.5 days after treatment relative to untreated fruit, *FcMADS6* expression was upregulated on day of harvest (3 days after treatment) following preharvest 1-MCP treatment. The decreased levels of *FcMADS8* transcript in treated fruit were still evident 3 days later, on day of harvest. With respect to the ethylene-synthesis pathway after storage, two *FcACS*s—*FcACS1L* and *FcACS3*—exhibited decreased transcript levels after storage and 2 days later, after shelf simulation. On the other hand, the expression levels of *FcACS2* and *FcACS4* peaked after storage. As for 1-MCP treatment effects on *FcACS* transcripts, expression of *FcACS2* and *FcACS4* was upregulated and that of *FcACS3* downregulated in treated versus untreated fruit 0.5 days after treatment. The upregulation of *FcACS2* transcription was restricted to the post-storage sample (10 days after treatment), while *FcACS4* upregulation was observed from day of harvest onwards. *FcACS3* expression did not change following 1-MCP treatment, in contrast to the evident expression peak on day of harvest in untreated fruit. Post-translational regulators of type 2 ACS proteins—*FcEOL1* and *FcEOL2*—showed different patterns following 1-MCP treatment (0.5 days after treatment) and in stored untreated fruit (10 days after treatment). *FcEOL1* expression increased very little in untreated fruit during the experiment. Following 1-MCP treatment, a sharp upregulation in *FcEOL1* transcription was observed and was still evident after storage. By comparison, *FcEOL2* transcription levels peaked on day of harvest in untreated fruit, and 1-MCP treatment temporarily downregulated this gene’s transcription (0.5 days after treatment). *FcACOL* expression underwent a moderate change in the untreated fruit following storage, whereas *FcACO2* expression decreased after storage. Compared to *FcACOL* transcription, which was not affected by 1-MCP treatment, *FcACO2* transcription was temporarily downregulated by the treatment, but its levels then increased, reaching those in the untreated fruit. The moderate increase observed in *FcACO3* expression in the untreated fruit after storage was greatly enhanced by 1-MCP application.

### Gene expression of ethylene-signal-transduction genes following preharvest 1-MCP treatment, storage, and shelf simulation

**Fig. 6.**
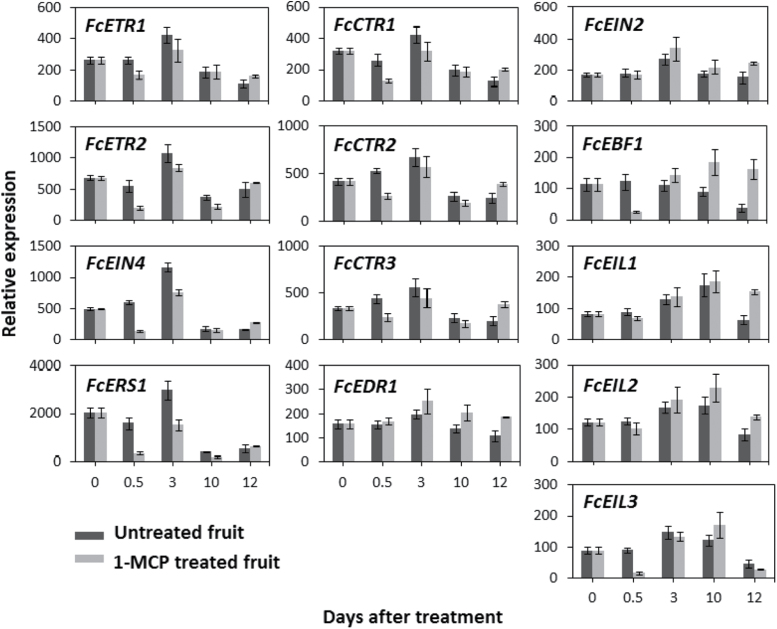
Expression patterns of ethylene-signal-transduction-related genes in preharvest 1-MCP-treated and untreated fruit (at ripening-onset fruit stage) followed by 1 week in cold storage and 2 days of shelf simulation. Ethylene-receptor genes (*FcETR1*, *FcETR2*, *FcEIN4*, and *FcERS1*), *CTR1-like* genes (*FcEDR1* and *FcCTR1–3*), *FcEIN2*, *FcEBF1*, and *EIN3/EIL* genes (*FcEIL1–3*). Days after treatment are as described in Fig. 1B. Experimental design is as described in Fig. 5.

To investigate the ethylene sensitivity of the ethylene-signal-transduction pathway in fig and its relationship with the improved storability of preharvest 1-MCP-treated fruit, expression of ethylene-signal-transduction genes was examined. Following storage (10 days after treatment) of untreated fruit (Fig. 1B), transcription levels of ethylene-receptor genes and *FcCTR*s were reduced (Fig. 6). These upstream signal-transduction components, except for *FcEDR1*, were downregulated following preharvest 1-MCP treatment (0.5 day after treatment). The downregulation effect of 1-MCP on *FcEIN4* and *FcERS1* expression was still evident on day of harvest (3 days after treatment), whereas *FcCTR1*, *FcCTR2*, and *FcCTR3* presented moderately higher levels in the treated versus untreated fruit after shelf simulation (12 days after treatment). As for *FcEDR1* expression, higher levels were found in 1-MCP-treated fruit after storage and shelf simulation relative to untreated fruit. The transcription pattern of *FcEIN2* was not significantly affected by 1-MCP treatment and generally presented a peak on day of harvest in both treated and untreated fruit; after storage, its expression level in treated fruit was moderately higher than in untreated fruit (1.5-fold change). Expression of *FcEBF1* showed a unique reaction to 1-MCP treatment: at 0.5 days after treatment, its expression level in the treated fruit was 5 times lower than in the untreated fruit, but after storage its expression level in the treated fruit was twice that in the untreated fruit. The upregulation of *FcEBF1* in treated fruit was even more pronounced after shelf simulation, when transcription in the treated fruit was 4.5 times higher than in the untreated fruit. *FcEIN2*, *FcEIL1*, and *FcEIL2* presented similar trends in treated and untreated fruit, with minor upregulation in treated fruit, compared to untreated, after shelf simulation. *FcEIL3*, by contrast, showed transient downregulation of transcription in treated fruit 0.5 days after treatment.

**Fig. 7.**
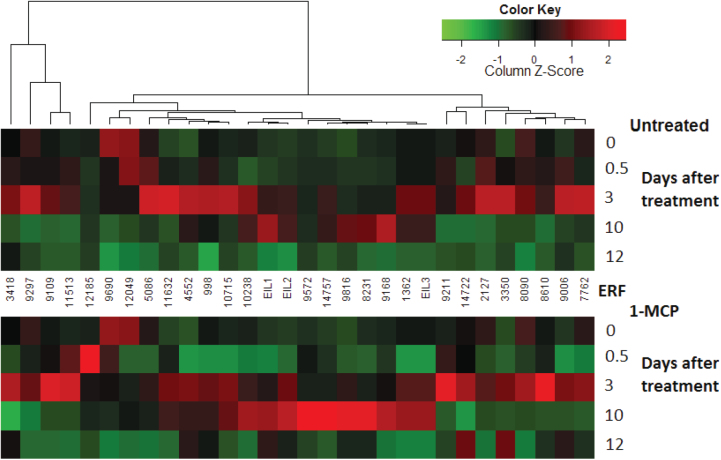
Expression patterns of ethylene-responsive factor genes (*ERF*s) in preharvest 1-MCP-treated and untreated fruit (at ripening-onset fruit stage) followed by 1 week in cold storage and 2 days of shelf simulation. Days after treatment are as described in Fig. 1B. Experimental design is as detailed in Fig. 5.

Given that *FcERF*s are downstream regulators of fruit ripening, which respond to ethylene by definition, their reaction to preharvest 1-MCP treatment was surprisingly mild (Fig. 7). Transcription of most of the *FcERF*s was temporarily downregulated by the treatment in fruit after on-tree overnight exposure (0.5 days after treatment) compared to untreated fruit. Transcription in the treated fruit was restored on day of harvest at the 50% purple stage. One exception was *FcERF12185*, whose transcription increased sharply in treated fruit (0.5 days after treatment) compared to untreated fruit. Its higher transcript level in treated fruit continued to day of harvest, but was downregulated to the level in untreated fruit after 1 week of storage. To associate *FcERF* expression patterns and *FcEIL* transcription levels following preharvest 1-MCP application, *FcEIL*s were clustered with *FcERF*s. Unlike the naturally on-tree-ripening fig, in which all *FcEIL*s clustered together, 1-MCP treatment led to branching of the *FcEIL*s into different clusters. *FcEIL3* was located in a different cluster than *FcEIL1* and *FcEIL2*, although the difference in expression patterns was moderate, because the difference between the clusters was moderate, as already noted.

## Discussion

The ripening process in fig fruit is categorized as climacteric, showing a rise in respiration rate and ethylene production at the onset of the ripening phase. Surprisingly, ripening-related ethylene production increases following pre- or postharvest 1-MCP application in an unexpected auto-inhibitory manner (Sozzi *et al.*, 2005; Owino *et al.*, 2006; Freiman *et al.*, 2012). This phenomenon supports the more recent notion that classification of fruits based on ethylene production is not very clear-cut (Paul *et al.*, 2012). Unlike most climacteric fruits, such as apple, *Pyrus communis* (pear), and banana, fig harvested before ripening onset will not ripen postharvest; therefore, commercial crops are harvested at the cultivar’s specific stage to reach ripe but not overripe stage (Flaishman *et al.*, 2008). In the current study, ethylene-related genes involved in the natural process of ripening in attached fruit were investigated. The analysis was expanded to preharvest 1-MCP-treated fruit at the ripening-onset stage and commercially mature fruit picked and stored following this treatment. The expression of fig *MADS-box* and ethylene-related genes was examined to understand their functions in fig fruit and to further explore ethylene- and ripening-regulation mechanisms in this unique fruit.

Members of the *MADS-box* gene family have been found to regulate ripening in several climacteric species: *RIN* and *TAGL1* in tomato, *PLENA* in *Prunus persica* (peach), *MADS1–5* in banana and *MADS8*, and *MADS9* in apple (Vrebalov et al., 2002, 2009; Itkin et al., 2009; Tadiello et al., 2009; Elitzur et al., 2010; Ireland et al., 2013). MADS-box protein activity is not restricted to ethylene-pathway regulation; direct targets of RIN, FUL1, and FUL2 in tomato include downstream metabolic genes, such as those from the carotenoid-synthesis pathway, as well as several transcription factors (Fujisawa et al., 2014). In addition, genes from the *MADS-box* family are involved in non-climacteric fruit ripening, such as *MADS9* in *Fragaria ananassa* (strawberry) and *CaMADS-RIN* in *Capsicum annuum* (pepper) (Seymour et al., 2011; Dong et al., 2014). In light of the central role of *MADS-box* genes in fruit ripening, eight *MADS-box* transcripts were previously identified and partially isolated from on-tree-developing fig fruit (Freiman *et al.*, 2014). In the present study, the transcripts of six of those genes were quantified to establish their function in fig fruit-ripening regulation. Among the examined *FcMADS-box* genes, the deduced amino acid sequences of *FcMADS6* and *FcMADS8* showed the highest homology to *SlRIN* among *FcMADS* genes (Freiman *et al.*, 2014). One of them, *FcMADS8*, presented increasing expression levels as ripening progressed in both the inflorescence and receptacle, resembling the pattern of *SlRIN* transcription in ripening tomato (Fig. 2; (Vrebalov et al., 2002; Martel et al., 2011; Yan et al., 2013)). Moreover, among the *FcMADS* genes with increasing transcription during fig ripening, inhibition of *FcMADS8* transcription by 1-MCP continued until 3 days after treatment (Fig. 5), indicating ethylene-induced behaviour similar to *SlRIN (Yan et al., 2013*). Considering the high resemblance of the predicted FcMADS8 protein to SlRIN (75% similarity, Supplementary Fig. S4), and the parallel expression patterns of these genes, including the prolonged ethylene sensitivity of *FcMADS8* transcription, this gene may well be the homologue of *SlRIN*, regulating the ripening process in fig fruit. As such, its downregulation following 1-MCP treatment could contribute to the improved storability of preharvest 1-MCP-treated fruit.

In climacteric fruits and senescing flowers, ethylene production is classified as system 2 autocatalytic ethylene synthesis (Yang and Hoffman, 1984). In the climacteric tomato model, ethylene-synthesis genes are associated with system 2 according to their upregulation during fruit ripening and downregulation in response to 1-MCP treatment (Yokotani et al., 2009; Van de Poel et al., 2012). This is also the case with ethylene-synthesis genes in apple (Yang et al., 2013). Surprisingly, in the climacteric fig, ripening-related ethylene production increased following pre- or postharvest 1-MCP application in an unexpected auto-inhibitory reaction. Fig ethylene-synthesis genes, three *ACS* genes, and one *ACO* gene were partially isolated by Owino *et al.* (2006), and their expression patterns were studied postharvest and following several treatments. All ethylene-synthesis genes in the study exhibited elevated transcript levels after harvest, while *FcACS2* expression was induced by postharvest 1-MCP treatment. To expand our knowledge of the function of ethylene-synthesis genes during fig ripening, expression patterns of four *ACS* genes and three *ACO* genes were examined (*FcACS4*, *FcACO2*, and *FcACO3* are newly presented here). All ethylene-synthesis genes seemed to be active during system 2 ethylene synthesis, increasing in the inflorescence during on-tree ripening (Fig. 2A). With the exception of *FcACO3*, expression of all of the genes increased in the receptacle as well, albeit to lower levels (Fig. 2B). Ethylene synthesis in fig, which is enhanced by 1-MCP treatment, can be related to *FcACS2*, *FcACS4*, and *FcACO3*, all of which were upregulated following treatment (Fig. 5). The unique combination of climacteric ripening alongside auto-inhibitory ethylene production has also been documented in postharvest ripening banana fruit (Inaba et al., 2007). The origin of the 1-MCP-enhanced ethylene levels was found to be the banana pulp; in the peel, ethylene production was inhibited by 1-MCP treatment. The expression pattern of *MaACS1* was found to correspond to the difference between the tissues’ ethylene reactions to 1-MCP. In addition, *MaACS1* was downregulated in the pulp once ethylene synthesis in the control fruit had started, as expected with this gene’s auto-inhibitory regulation. It is tempting to assume that, in fig, ethylene synthesis is differentially affected by 1-MCP in the inflorescence and receptacle. That said, ethylene-synthesis genes in the two tissues only showed distinct magnitudes of otherwise similar expression patterns in on-tree-ripening figs (Fig. 3). Moreover, examination of ethylene-synthesis genes in separate tissues following preharvest 1-MCP application showed that upregulation of ethylene-synthesis genes is not restricted to either tissue (data not shown). In light of these findings, the duality of the ethylene characteristics in fig is suspected to be a consequence of an overcomplicated feedback mechanism that is not specific to certain genes, or tissues. Ethylene synthesis is also influenced by ETO1 and EOL proteins that regulate type 2 ACSs post-translation (McClellan and Chang, 2008). Here, *FcEOL1* and *FcEOL2* were identified in addition to *ACS* and *ACO* genes. According to their expression patterns, these genes may serve as ethylene-synthesis regulators in ripening fruit but not in relation to the ethylene auto-inhibitory mechanism (Figs. 3 and 6). In fact, tomato *ETO1* expression is restricted to the fully ripe fruit stage, whereas in fig both genes are active during the ripening period (Fig. 2), presumably regulating the type 2 ACSs *FcACS3* and *FcACS4*. To the best of our knowledge, this is the first example of *EOL* genes that are active as fruit ripening progresses.

Compared to ethylene synthesis, ethylene-signal transduction is much more complex. Expression patterns of tomato ethylene receptors during fruit ripening have shown that three out of seven receptors have increased transcription levels in parallel to increasing levels of ethylene (Kevany et al., 2007). Because ethylene receptors function as ethylene-response inhibitors (Kevany et al., 2007), the elevation in receptor expression during ripening presents a feedback inhibitory mechanism of the hormone by signal-transduction components. As such, silencing of two of these receptors with a fruit-specific promoter causes an early-ripening phenotype (Kevany et al., 2007, 2008). Like ethylene receptors, CTRs function as ethylene-response inhibitors and their transcription induction during tomato ripening implies a role in the feedback inhibitory mechanism of that process (Merchante et al., 2013). The protein CTR phosphorylates EIN2 to inactivate it, while EIN2 is a positive regulator of ethylene response (Ji and Guo, 2013). In tomato, expression of *EIN2,* the only gene of its kind, increases at the onset of ripening (Gapper et al., 2013). In *Arabidopsis*, EIN2 is a target for protein turnover via the proteosome (Qiao et al., 2009) though, to date, no homologues for its targeting proteins have been found in tomato. Another downstream positive ethylene-response regulator is EIN3; as such, antisense suppression of tomato *EIL1*, *EIL2*, and *EIL3* reduced ethylene sensitivity (Tieman et al., 2001). Positively regulating the ethylene response, *EIL* genes are upregulated in ripening tomato (Yokotani et al., 2003). Like EIN2, EIN3 is targeted for protein turnover; thus, repression of SlEBF1/SlEBF2—the EILs’ targeting proteins—resulted in a constitutive ethylene response and early ripening (Yang et al., 2010). The last step in the ethylene-signal-transduction pathway is *ERF* activation by EIN3/EILs. The *ERF* family is large, comprising both repressors and activators, with a degree of functional redundancy among its members (Klee and Giovannoni, 2011; Licausi et al., 2013). On the one hand, SlERF1 positively regulates fruit ripening and softening in tomato fruit; on the other hand, SlERF6 has been characterized as a negative regulator of ethylene and carotenoid biosynthesis, while SlERF.B3 has contrasting effects on tomato-ripening processes (Li et al., 2007; Lee et al., 2012; Liu et al., 2014). These studies demonstrate the balancing role of the *ERF* family, contributing to the complexity of ripening regulation in climacteric fruit. The *ERF* family will be discussed separately further on.

This study represents a first attempt to follow the expression patterns of a large group of genes involved in the fig’s ethylene-signal-transduction cascade. In general, fig ethylene-signal-transduction genes presented expression patterns similar to those in tomato and apple during ripening (Klee and Giovannoni, 2011; Yang et al., 2013). However, this resemblance was restricted to the inflorescence (Fig. 3A). In the receptacle, minor changes compared to those in the inflorescence resulted in opposing patterns in most genes (Fig. 3B). The correspondence between ethylene-signal-transduction genes in the fig inflorescence and tomato suggests a similar feedback mechanism for the hormone by its receptors and *FcCTR* components. Ethylene-signal transduction in fig is probably subjected to negative post-translational regulation by *FcEBF1*, as presented here. *FcEBF1* expression levels rose in the fig inflorescence at the ripe stage, presumably to negatively control FcEIL proteins (Fig. 3A). Interestingly, this gene was downregulated following preharvest 1-MCP treatment, although it was upregulated again after storage (Fig. 6). *FcEBF1* is assumed to be one of the key genes responsible for the improved storability conferred by preharvest 1-MCP application, along with *FcEIL3*, which is the only *FcEIL* gene that was downregulated following preharvest 1-MCP treatment (Fig. 6). Regarding the effect of preharvest 1-MCP treatment on ethylene production, the mentioned upstream members of the signal-transduction pathway (receptors, *CTR*s, *EIN2*, *EBF1*, and *EIL*s) give few clues to the fig’s specific regulatory network. Downregulation of ethylene-receptor and CTR transcription is evident in 1-MCP-treated tomato and apple, both attached and detached, with both fruit showing the classical climacteric autocatalytic ethylene reaction, unlike the ambiguous reaction of fig fruit to 1-MCP (Varanasi et al., 2013; Yan et al., 2013; Yang et al., 2013). The auto-inhibitory nature of ethylene in ripening fig may therefore be a consequence of the downstream regulators of ethylene-signal transduction—the *ERF*s.

As noted above, the *ERF* family is highly complex, contributing to the fine regulation of climacteric fruit ripening. In the *FcERF*s analysis during on-tree-ripening, cluster 1 consists of *FcERF*s that regulate processes in the receptacle towards ripening onset, but not during the ripening period. Cluster 2 only contains *FcERF8231*, regulating processes towards the commercially ripe stage in both receptacle and inflorescence. Clusters 3 and 8 represent *FcERF*s controlling activities in the inflorescence after the initiation of fig colour change, whereas cluster 4 exhibited this trend in the receptacle. Clusters 5 and 7 are more active in the receptacle than in the inflorescence, while cluster 6 *ERF*s regulate processes towards ripening completion in the inflorescence, but in the receptacle this cluster is active at the yellow and 50% purple stages. The fact that all three *EIL*s were designated to a single cluster may indicate the existence of additional *EIL* genes in the ripening fig or regulation that includes more than one component in a complex cascade. Only one *FcERF*, namely *FcERF12185*, was upregulated in response to preharvest 1-MCP treatment in yellow-stage figs, while its transcripts remained at higher levels than in the untreated fruit on harvest day, 3 days after treatment (Fig. 7). This *ERF* may well be responsible for the burst in ethylene synthesis in treated fruit following treatment, targeting ethylene-synthesis genes as well as activating other positive regulators of ethylene synthesis, resulting in higher ethylene levels after storage and shelf simulation. In the fruit that ripened naturally on the tree, *FcERF12185* transcription only increased at the fully ripe stage, meaning that it is probably not responsible for the climacteric ethylene rise or metabolic ripening processes at early ripening stages. It may be associated with the regulation of system 1, rather than system 2 ethylene synthesis. None of *FcERF12185* homologs found have specific functions related to fruit development, ethylene regulation, or otherwise (through BLASTX against the nr collection in NCBI, data not shown). Since ERF protein structures are diverse and their action depends on the promoter sequences of their target genes, further investigation of this unique *FcERF*, its targets, and its function should unravel its role in the non-climacteric behaviour of the ‘climacteric’ fig fruit. Regarding the temporary downregulation of most *FcERF*s following preharvest 1-MCP treatment (Fig. 7), one might ask whether this minor difference from the untreated fruit is responsible for the high quality of treated stored fruit. Though some *FcERF*s reach high expression levels after storage (Fig. 7), it is proposed that the ripening-retarded fig subjected to storage may be less vulnerable to storage damage than the untreated fruit, in which tissues are in a more advanced stage of ripening. As such, untreated fruit have a shorter shelf-life and their storability is lower.

The different structures of fleshy fruits are defined according to the flower organ’s fate in the developing fruit (Esau, 1977). The tomato fruit is an example of a true fruit, developed from the ovary with no accessory tissue. The fig, on the other hand, is an accessory multiple fruit composed of individual drupelets developed from the ovaries in a closed inflorescence, the syconium (Fig. 1C). The surrounding receptacle, which constitutes a large portion of the edible fruit flesh, is the visible part of the enclosed inflorescence/fruit as the syconium develops (Condit, 1947). A few studies have shown that the molecular events regulating ripening processes exhibit different profiles in the different tissues of accessory fruits. In apple, *MADS-box* and ethylene-synthesis genes present different transcription levels between the core and the cortex, and the same phenomenon has been documented for *MADS-box* gene expression in pear (Ireland et al., 2013; Ubi et al., 2013). Diverse gene-expression patterns in banana peel and pulp were mentioned above and even in ripening tomato fruit, tissue-specific trends of ethylene-synthesis activity have been recently found (Van de Poel et al., 2014). In this study, expression trends of most of the examined genes were dissimilar in the fig inflorescence and receptacle. In the inflorescence, as mentioned, *FcMADS-box* genes as well as ethylene-synthesis and signal-transduction genes showed patterns characteristic of climacteric fruits. In the receptacle, this was only true for *FcMADS8* and the ethylene-synthesis genes (with the exception of *FcACO3*). That said, the fig drupelets developed inside the syconium are proposed to function as parthenocarpic true fruit, regulating ripening processes for the whole accessory fruit. As such, the inflorescence may produce higher ethylene levels than the receptacle. Given that ethylene can diffuse freely between cells, it is assumed that the activity of *FcMADS8* and ethylene-synthesis genes in the receptacle is a reaction to the ethylene produced in the inflorescence at ripening onset.

**Fig. 8.**
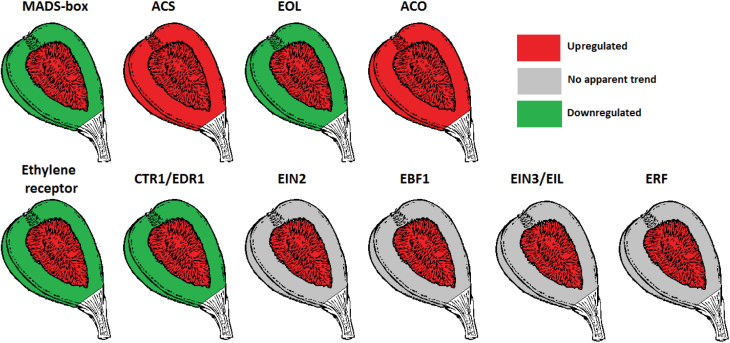
Schematic representation of the different gene families’ activities in fig fruit during on-tree ripening. The general expression pattern was concluded from the majority of genes in a family that were up- or downregulated during the ripening process. No apparent trend was defined when expression patterns showed combined trends in one gene family or a changing pattern with ripening.

**Fig. 9.**
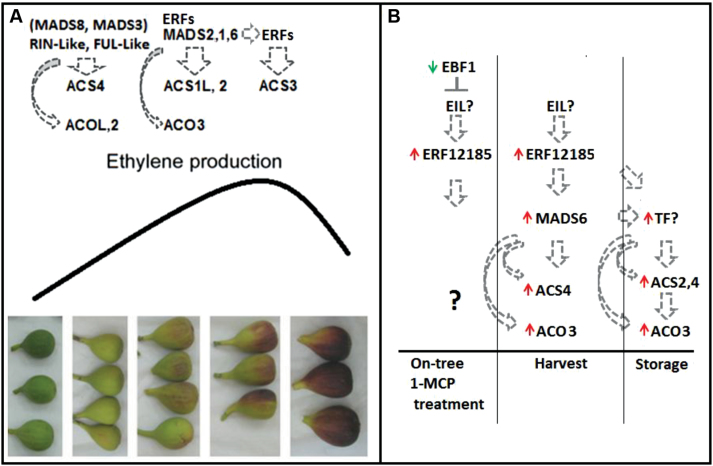
Proposed model of ethylene-regulation and synthesis genes in fig fruit. (A) Proposed ethylene-regulator and ethylene-synthesis gene activity during on-tree ripening. (B) Proposed ethylene-regulation and ethylene-synthesis gene activity following on-tree 1-MCP treatment of ripening-onset fruit, in commercially mature fruit harvested 3 days after treatment, and in stored fruit. Red arrow, upregulated genes in treated fruit compared to untreated fruit; green arrow, downregulated genes in treated fruit compared to untreated fruit.

To conclude, the expression patterns of ethylene-synthesis and signal-transduction genes in fig were similar to those in tomato and apple during ripening, specifically in the fig inflorescence–drupelet section, as summarized in Fig. 8. *FcMADS8* shares several features with the well-studied *SlRIN*; as such it is a potential key regulator of ripening onset and events. The auto-inhibition reaction of ethylene production may be related to the direct functions of *FcACS2*, *FcACS4*, and *FcACO3*, as detailed in Fig. 9. Genes of the ethylene-signal-transduction cascade in the fig inflorescence present expression patterns similar to those in tomato and apple during ripening, suggesting a similar feedback mechanism of the hormone by its negative regulators of signal transduction. *FcMADS8*, *FcEBF1*, and *FcEIL3* are proposed to be the key genes responsible for the improved storability of preharvest 1-MCP-treated fruit. Several *FcERF*s were also shown to be suppressed following 1-MCP treatment. In addition to the association of ethylene-synthesis genes to the ethylene profile during ripening and following 1-MCP application, a possible regulator of the feedback reaction is proposed, namely *FcERF12185* (Fig. 9B). This downstream component of ethylene-signal transduction could play a role in regulating ethylene-synthesis system 1 in reaction to 1-MCP, causing the non-climacteric behaviour of fig ethylene production.

## Supplementary material

Table S1. Fig genes homologous to *MADS-box*, ethylene-synthesis, and ethylene-signal-transduction genes subjected to gene-expression analysis.

Table S2. Primers used for transcript isolation and sequencing.

Table S3. Primers used for high-throughput real-time quantitative PCR.

Fig. S1. Newly isolated *FcACS1L* transcript alignment with the published sequence of *FcACS1*.

Fig. S2. Phylogenetic analysis of *FcACS* predicted proteins.

Fig. S3. Isolated *FcACOL* transcript (published previously under NCBI accession number AB307720.1 2007) aligned with the published sequence of *FcACO1* (Owino *et al.*, 2006).

Fig. S4. Alignment of deduced FcMADS8 amino acid sequence and SlRIN protein.

Supplementary Data
