# Supplementary material for: The ambiguous ripening nature of the fig (Ficus carica L.) fruit: a gene-expression study of potential ripening regulators and ethylene-related genes
Source: J Exp Bot. 2015 May 8;66(11):3309–24. doi: 10.1093/jxb/erv140 (PMC4449545; doi:10.1093/jxb/erv140)
Supplement: Supplementary Data [file supp_66_11_3309__index.html]

The ambiguous ripening nature of the fig (Ficus carica L.) fruit: a gene-expression study of potential ripening regulators and ethylene-related genes — The ambiguous ripening nature of the fig (Ficus carica L.) fruit: a gene-expression study of potential ripening regulators and ethylene-related genes — Supplementary Data 

# The ambiguous ripening nature of the fig (*Ficus carica* L.) fruit: a gene-expression study of potential ripening regulators and ethylene-related genes

## Supplementary Data

Data files

**Files in this Data Supplement:**

- Supplementary Data - Supplementary Data
