# Supplementary material for: The ambiguous ripening nature of the fig (Ficus carica L.) fruit: a gene-expression study of potential ripening regulators and ethylene-related genes
Source: J Exp Bot. 2015 May 8;66(11):3309–24. doi: 10.1093/jxb/erv140 (PMC4449545; doi:10.1093/jxb/erv140)
Supplement: Supplementary Data [file supp_erv140_jexbot143974_file001.pdf]

Supplementary material for the manuscript:

The ambiguous ripening nature of the fig (*Ficus carica* L.) fruit: a gene-expression study of potential ripening regulators and ethylene-related genes

Zohar E. Freiman[1,2], Yogev Rosianskey[1,2], Rajeswari Dasmohapatra[1], Itzhak Kamara[1] and Moshe A. Flaishman[1,*]

[1] Institute of Plant Sciences, Agricultural Research Organization, P.O. Box 6, Bet-Dagan 50250, Israel
[2] The Robert H. Smith Institute of Plant Sciences and Genetics in Agriculture, Faculty of Agriculture, Food and Environment, The Hebrew University of Jerusalem, P.O. Box 12, Rehovot 76100, Israel
* To whom correspondence should be addressed. E-mail: vhmoshea@agri.gov.il

Table S1. Fig genes homologous to *MADS-box*, ethylene-synthesis and ethylene-signal-transduction genes subjected to gene-expression analysis. Homologous genes from *Arabidopsis*, tomato and apple are noted.

**Gene name (sequence ID of cDNA isolates)**	**Arabidopsis homologue (Protein sequence ID, NCBI)**	**Tomato homologue (Protein sequence ID, NCBI)**	**Apple homologue (Protein sequence ID, NCBI)**
FcMADS2 (KJ506153)	AP1 (NP_177074.1)	MADS-MC (NP_001234665.1)	MADS5 (ABG85297.1)
FcMADS3 (KJ506154)	FUL, AGL8 (NP_568929.1)	FUL1 (AAP83379.1)	MADS2.1 (ABB22023.1)
FcMADS4 (KJ506155)	SVP (BAD43004.1)	JOINTLESS-like (XP_004250145.1)	JOINTLESS (ABD66219.2)
FcMADS5 (KJ506156)	SEP3 (NP_850953.1)	AGL31-Like (XP_004239235.1)	MADS9 (CAA04920.1)
FcMADS6 (KJ506157)	AGL2 (AAA32732.1)	MADS1 (NP_001234380.1)	MADS3 (AAD51422.1)
FcMADS8 (KJ506159)	SEP1 (NP_568322.1)	RIN (NP_001234670.1)	MADS4 (AAD51423.1)
FcACS1L	ACS6 (NP_192867.1)	ACS1B (AAF97615.1)	ACS-5B (BAA92351.1)
FcACS2 (DQ269493.1)	ACS1 (NP_191710.1)	ACS1A (AAF97614.1)	ACS-5B (BAA92351.1)
FcACS3 (DQ269494.1)	ACS8 (NP_195491.1)	ACS8 (AAQ13597.1)	ACS-1 (AAA73941.1)
FcACS4	ACS8 (NP_195491.1)	ACS3 (AAA78789.1)	ACS-1 (AAA73941.1)
FcACOL (AB307720.1)	ACO4 (NP_171994.1)	ACO1 (CAA41212.1)	ACO1 (AAC36461.1)
FcACO2	ACO5 (NP_565154.1)	ACO5-Like (XP_004242046.1)	ACO2 (AAB94031.1)
FcACO3	ACO1 (NP_179549.1)	ACO5 (NP_001234037.1)	ACO2 (AAB94031.1)
FcEOL1	ETO1 (AAT01656)	ETO1-Like	Low homology

		(XP_004247013.1)	
FcEOL2	ETO1-Like 1 (NP_192177.1)	EOL1 (AAZ08351.1)	Low homology
FcETR1	ETR1 (NP_176808.3)	ETR1 (NP_001234149.1)	ETR1b (AAW69924.1)
FcETR2	ETR2 (NP_188956.1)	ETR4 (NP_001234205.1)	ETR2 (ABI58286.1)
FcEIN4	EIN4 (NP_187108.1)	ETR5 (NP_001234212.1)	ETR2 (ABI58286.1)
FcERS1	ERS1 (NP_181626.1)	Nr (NP_001233894.1)	ERS2 (BAE97296.1)
FcCTR1	CTR1 (NP_195993.1)	CTR3 (NP_001234454.1)	CTR1 (ABI58288.1)
FcCTR2	CTR1 (NP_195993.1)	TCTR1 (NP_001234330.1)	CTR1 variant (ABI58289.1)
FcCTR3	CTR1 (NP_195993.1)	CTR3 (NP_001234454.1)	CTR1 (ABI58288.1)
FcEDR1	EDR1 (ABR45974.1)	CTR2 (NP_001234768.1)	CTR2 (ABI33217.1)
FcEIN2	EIN2 (NP_195948.1)	EIN2 (NP_001234518.1)	EIN2 (ABI33219.1)
FcEBF1	EIN3-binding F-box protein 1 (NP_565597.1)	EIN3-binding F-box protein 1 (NP_001234858.1)	EIN3 binding F-box 1 (AFT92041.1)
FcEIL1	EIL3 (NP_177514.1)	EIL3 (NP_001234546.1)	EIL1 (ADE41153.1)
FcEIL2	EIN3 (NP_188713.1)	EIL3 (NP_001234546.1)	EIL2 (ADE41154.1)
FcEIL3	EIN3 (NP_188713.1)	EIL3 (NP_001234546.1)	EIL3 (ADE41155.1)
FcERF9168	ERF071 (NP_182274.1)	ethylene response factor 2 (NP_001234308.1)	ERF071-like (XP_008386662.1)
FcERF12185	ethylene-responsive transcription factor TINY (NP_197953.1)	dehydration-responsive element-binding protein 3-like (XP_004241746.1)	AP2 domain class transcription factor (ADE41141.1)
FcERF9211	ERF118 (NP_177022.1)	ERF118-like isoform 1 (XP_004238935.1)	ethylene-responsive transcription factor CRF6-like (NP_001280962.1)
FcERF9297	ERF118 (NP_177022.1)	ERF118-like isoform 1 (XP_004238935.1)	ERF118-like (XP_008368213.1)
FcERF8231	ethylene-responsive	RAP2.6-like protein	AP2 domain class

	transcription factor ABR1 (NP_201280.1)	(ABY57638.1)	transcription factor (ADE41125.1)
FcERF5086	ERF061 (NP_176620.1)	ERF061-Like (XP_004245520.1)	ERF061 (XP_008341502.1)
FcERF9572	ERF109 (NP_195167.1)	RAP2.6-like protein (ABY57638.1)	AP2 domain class transcription factor (ADE41151.1)
FcERF9690	ethylene responsive element binding factor 1 (BAA32418.1)	ethylene-responsive transcription factor 1-like (XP_004235186.1)	ethylene-responsive transcription factor 2-like (XP_008352246.1)
FcERF9109	ERF025 (NP_200015.1)	ERF025-like (XP_004240742.1)	ERF027-like (XP_008381395.1)
FcERF11513	dehydration-responsive element-binding protein 1F (NP_172721.1)	dehydration-responsive element-binding protein 1C-like (XP_004234351.1)	AP2 domain class transcription factor (ADE41100.1)
FcERF11632	dehydration-responsive element-binding protein 1D (NP_200012.1)	dehydration-responsive element-binding protein 1C-like (XP_004234351.1)	AP2 domain class transcription factor (ADE41100.1)
FcERF12049	ethylene-responsive transcription factor (NP_188965.1)	ethylene responsive element binding protein (NP_001234695.1)	AP2 domain class transcription factor (ADE41122.1)
FcERF8090	ethylene-responsive transcription factor CRF4 (NP_194524.2)	pathogenesis-related genes transcriptional activator PTI6 (NP_001233991.1)	pathogenesis-related genes transcriptional activator PTI6-like (XP_008384656.1)
FcERF10238	ethylene-responsive transcription factor 9 (NP_199234.1)	ethylene response factor 3 (AAO34705.1)	ethylene-responsive transcription factor 4 (XP_008371404.1)
FcERF10715	ethylene-responsive transcription factor 9 (NP_199234.1)	ethylene-responsive transcription factor 4-like (XP_004243371.1)	ethylene-responsive transcription factor 4-like (XP_008358676.1)
FcERF998	ethylene-responsive transcription factor RAP2-2 (NP_850582.1)	transcription factor JERF1 (NP_001234513.1)	ethylene-responsive transcription factor RAP2-12-like (NP_001280975.1)
FcERF14722	dehydration-responsive	dehydration-responsive	putative dehydration-

	element-binding protein 2A (NP_001031837.1)	element-binding protein (NP_001234689.1)	responsive element-binding protein 2H (XP_008362249.1)
FcERF2127	ERF060 (NP_195688.1)	ethylene-responsive transcription factor RAP2-4-like isoform 1 (XP_004237407.1)	ERF060 (NP_001288047.1)
FcERF9816	ethylene-responsive transcription factor RAP2-1 (NP_564496.1)	ERF010-like (XP_004238136.1)	ERF011-like (XP_008378018.1)
FcERF14757	ERF053 (NP_179685.1)	ERF054-like (XP_004243339.1)	ERF054-like (XP_008342928.1)
FcERF8610	putative AP2 domain transcription factor (AAM65663.1)	AP2-like ethylene-responsive transcription factor At2g41710-like (XP_004231573.1)	AP2-like ethylene-responsive transcription factor At2g41710 (XP_008387305.1)
FcERF3350	ERF106 (AAK97736.1)	ethylene response factor 4 (NP_001234313.1)	ERF106-like (XP_008376371.1)
FcERF9006	ERF114 (NP_200995.1)	ERF115-like (XP_004235847.1)	ERF114-like (XP_008356833.1)
FcERF1362	ethylene-responsive transcription factor RAP2-3 (NP_188299.1)	ERF6 (NP_001266125.1)	ethylene-responsive transcription factor RAP2-3-like (NP_001287809.1)
FcERF7762	ethylene-responsive transcription factor 3 (NP_175479.1)	ethylene-responsive transcription factor 3-like (XP_004248374.1)	ethylene-responsive transcription factor 3-like (XP_008362296.1)
FcERF4552	putative AP2/EREBP transcription factor (AAT44938.1)	ERF062-like (XP_004247959.1)	ERF062 (XP_008380561.1|)
FcERF3418	ERF003 (NP_197901.1)	ethylene response factor 1 (NP_001234848.1)	ERF003-like (XP_008378704.1)

Table S2. Primers used for transcript isolation and sequencing. All primers except *FcACS1L*-Forward* were designed according to the developing fig transcriptome. *Designed according to GenBank sequence DQ269492.1.

**Gene name cDNA isolates**	**Forward primer (3'→5')**	**Reverse primer (3'→5')**
FcACS1L	ATGGGTTTGGCGGAGAAC*	TTAAGTTCTTGCTTGGACTAAAGGTGATTG
FcACS4	TTCTCCGGCACCGTCTTTGACTCC	TTAACGGTCGGTTTCACGATCATCCGAT
FcACO2	ATGGCAATCCCAGTGATTGATTTCTCAAAGC	CTACATGGCTCTGACAGCTTGGAACCT
FcACO3	ATGGCTCTCCTACACCAAGCTTGC	CTAGATGACCGTTGCTCCCATTGACCAT

Table S3. Primers used for high-throughput real-time qPCR.

Gene name	Forward primer (3'→5')	Reverse primer (3'→5')
FcMADS2	GTATGGCGGTAGAGCATCAGATGC	GTAAGTTCCTCCAATGTTTAAGCAGGGAAG
FcMADS3	CTCATACTGCGGAGGCTTCTCAATC	GCATCCAGGGTGGTATGCAAGTG
FcMADS4	AGACTCAGGTGAACAAGGCCAGTCA	CAACTTGAGAGCGGTATCCGAGC
FcMADS5	GCTCTTGTCATGGACCAAGCTGG	GCGGCAGTTAATCCCATTCGCTATG
FcMADS6	TTCACCCGCCATCCTACTCAACC	CTCCAACATTCATCCCATCTGGACC
FcMADS8	GTAAGTGAAGCTGCTAGGGAGCATAA	AGTGCGTTATTGCATGGTAAAGGCTC
FcACS1L	CGAGAGCGCTAGAAGATTGG	CTGTGCAAATCCATCCACAA
FcACS2	GTCGAGCTTCGGACTAGTGTC	CCTCTTCGAGCTCTCCGA
FcACS3	TCGTAAGCGTCATGGAGATTC	TCGTAAGCGTCATGGAGATTC
FcACS4	GCCTTTGCGGAAACCTTAAT	TGCTAATTGGAACACCCATCT
FcACOL	GAGCAGGAAACCAAAGTGGT	CCTTCATGGCTTCAAACCTAG
FcACO2	CAAATCCTGAAAGGCGAGAAG	GTGCCAAACACTCTTGTACCTC
FcACO3	GTTTGCAGTTCCTCAAAGATGA	TCCATTGCTCAGAATTTCCAC
FcEOL1	GTGGTATTGACGTTAGAACCCAA	TTACTTAATTGTCGAACCATACGG
FcEOL2	GACCTGGAGATGGTTACCCAA	TGCTTCAGTTTCCTTCTGGTT
FcETR1	GCAATCTGCAAGAGGTTCGT	ATGGAGCTTCGATTCATTGG
FcETR2	GAATTCGTTCCAGAGGGTGA	ACCATCGCCTCGACAATAAC
FcEIN4	CAATGCGAATCCGGAAGTT	GAAGTTCATCCGCCAAACCT
FcERS1	ACCTGCCCTTTAGCCAGAAT	CAAAACCATGATCGCATAGC
FcCTR1	GGACCTCCCACTGATGAAGA	GACATGTCATCACCGTGACC
FcCTR2	CAGCTCAGGTTGTAGCAGCA	CCAGCAGATTTTGATTAAGGC
FcCTR3	TTGGCTACATGCTGGAATGA	CACGATGGACACAAAATCCA

FcEDR1	TGGCTAGGGGTATGAATTGC	TCACCAACCTTGACATTCCA
FcEIN2	CTATACCTGATTCACACACCGAC	CATGGGCTTTACTACGACAGG
FcEBF1	TTAGCCAAGCAGGGATCGT	TGAGCGTGGGTACACCAGA
FcEIL1	GGAGGGTCCAACCTATGGAT	GTGATGGCTGAGGCAGTGTA
FcEIL2	GGTTGTTATGGGAGGTAATATCC	GTCCGCAATATTGTCATTGG
FcEIL3	CGGTAGTGGCTCCTAAGATTAAA	AATCCTGATAGGGGCATTGA
FcERF9168	TTGTGTTGGAAGAGGCAGAG	CCGTCCAGGTACGGAATCT
FcERF12185	GAGTCGATCGGGAGTGAGTT	CTCGCCGTAAAAGTACTCATCA
FcERF9211	ACTAGTTTCGCCGCTAGCAA	ACTAGTTTCGCCGCTAGCAA
FcERF9297	GCAGCATTGACGATGTTCAG	GCAAGGAATGTTGAGAGCTT
FcERF8231	CTTTTTGAAGGTGGCGAACC	TTGATTCTGGGAGGCTCTTC
FcERF5086	CACCGTCCTACGTTTCCAAT	AACCTCCCAAATCAAATCAGG
FcERF9572	GAAGAAGAATTACAGAGGAGTGAGG	GCCGTGTTAAAAGTCCCG
FcERF9690	ACGGAGAGAGCACGATGG	CTGAACCGGAATTCGTGACT
FcERF9109	GCCGGAGGAGAGTTTATTGA	ACGACGGCGTATTGATCCT
FcERF11513	TGGGAGGAGGGTGTTCAA	CTTGTTCGGCTCACGGAG
FcERF11632	GGGAGGAGGGTATTCAAGGA	GACTTGGTATTCGGCTCTCG
FcERF12049	GTGGGAGAGTTTTGATTTTTCC	TTCTCGGTTCGTCTCGGA
FcERF8090	GAGTCAGACATCTCTTGCCG	AACCAGACCCGCTTTCTC
FcERF10238	GTTAGATTCCCGTTCCAGCA	GTTGATACGGGACGCTCTTG
FcERF10715	GCTTAGCAGACCGGATTTCA	TTCAACCACGGAAGACGAGT
FcERF998	ATGCACCACTGGATGCCTT	AGGAAAGCAGGGGAACTAGC
FcERF14722	GAATTCTCGTTGTAGCTTTAGGG	GTCGGGAAGGTGCCTAGC
FcERF2127	ATATGGTCATGTCAACCGCA	GGTTTTGAATTAGCACCAACG
FcERF9816	CGAACAAGAGGTCGAGGATT	AAGATGAGGTCGGGGAAGTT

FcERF14757	CAGCCCATCAACACGACC	CTAGCCAGAGGCGAGTCCT
FcERF8610	ACGATGGTGGAAGTGAGAGG	TTGGTTAGCAATGGTGCAGAC
FcERF3350	CGCATCCGAACCCAATTC	AATCTTTCCGTGGCTCAGATC
FcERF9006	CGGACCTCTACCACTATGCAC	TGTAATGAAAGGGAGCTGCTG
FcERF1362	TGTCGCCACCTAACAAAGTG	TGTTCTTGCGAGTTCGTTGA
FcERF7762	CGGTCCAAGAAGTAAGATTCAGA	GAAGGTGCCGAGCCAGA
FcERF4552	AAGGCGTCTCGGGTTCTC	AAACCATTGGCTTTCGAGTC
FcERF3418	CGAAACCCGTCGAAACG	GGTTGCTCTCAACTTTAACCTTC

Fig. S1. Newly isolated *FcACS1L* transcript alignment with published sequence of *FcACS1*, DQ269492.1 (Owino *et al.*, 2006). The frame-shift position is marked by a red asterisk. Alignment was performed by MUSCLE program using default parameters (http://www.ebi.ac.uk/Tools/msa/muscle) (Edgar, 2004).

```
FcACS1 DQ269492.1   1     ATGGGTTTGGCGGAGAACCAGCTCTGCTTTGATTTGGTTCAAGACTGGATTCTGAACAAC  60
FcACS1L             1     ATGGGTTTGGCGGAGAACCAGCTCTGCTTTGATTTGGTTCAAGACTGGATTCTGAACAAC  60

FcACS1 DQ269492.1   61    CCCAGAGCCTCCATTTGCACGCCTGAAGGAGTTGATGAATTCAGAGACATAGCTATTTTC  120
FcACS1L             61    CCCAGAGCCTCCATTTGCACGCCTGAAGGAGTTGATGAATTCAGAGACATAGCTATTTTC  120

FcACS1 DQ269492.1   121   CAAGACTACCATGGCCTGCCAGAGTTCCGAAAAGCTGTAGCGGAGTTCATGGCACAGGTT  180
FcACS1L             121   CAAGACTACCATGGCCTGCCAGAGTTCCGAAAAGCTGTAGCGGAGTTCATGGCACAGGTT  180

FcACS1 DQ269492.1   181   AGAGGAAACCGAGTAACTTTCGATCCCGACCGCATTGTAATGAGCGGAGGAGCGACCGGT  240
FcACS1L             181   AGAGGAAACCGAGTAACTTTCGATCCCGACCGCATTGTAATGAGCGGAGGAGCGACCGGT  240

FcACS1 DQ269492.1   241   GCTCATGAGATGGTTACTTTTTGCTTGGCTGATCCAGGAGAAGCATTTCTGGTTCCTGTT  300
FcACS1L             241   GCTCATGAGATGGTTACTTTTTGCTTGGCTGATCCAGGAGAAGCATTTCTGGTTCCTGTT  300

FcACS1 DQ269492.1   301   CCTTATTATCCAGGATTCAACCGAGACTTGAGATGGAGAACGGGAGTACAACTAATTCCG  360
FcACS1L             301   CCTTATTATCCAGGATTCGACCGAGACTTGAGATGGAGAACGGGAGTACAACTAATTCCG  360

FcACS1 DQ269492.1   361   GTCCACAGCGAAAGCGCAAACAACTTCAAGGTGACAAGGGAAGCCTTGGAAGAAGCCTAT  420
FcACS1L             361   GTCCACAGCGAAAGCGCAAACAACTTCAAGGTGACAAGGGAAGCCTTGGAAGAAGCCTAT  420

FcACS1 DQ269492.1   421   GAGAAGGCTCAAGAGGAGAACATAAGAGTGAAAGGTTTGCTGATCACCAATCCATCAAAC  480
FcACS1L             421   GAGAAGGCTCAAGAGGAGAACATAAGAGTGAAAGGTTTGCTGATCACCAATCCATCAAAC  480

FcACS1 DQ269492.1   481   CCCTTGGGCACCATCCTAGACAGAGAGACACTAAGGATGATAGTGCGGTTCATCAACGAG  540
FcACS1L             481   CCCTTGGGCACCATCCTAGACAGAGAGACACTAAGGATGATAGTGCGGTTCATCAACGAG  540

FcACS1 DQ269492.1   541   AAGAGCATCCACCTAGTCTGCGACGAAATCTATGCTGCCACGGTCTTCAACCAGCCGCCA  600
FcACS1L             541   AAGAGCATCCACCTAGTCTGCGACGAAATCTATGCTGCCACGGTCTTCAACCAGCCGCCA  600

FcACS1 DQ269492.1   601   TTCGTGAGCATCTCGGAGATCATAGCAGAGGATACAGACATCGAGTGCCACACAGACCTC  660
FcACS1L             601   TTCGTGAGCATCTCGGAGATCATAGCAGAGGATGCAGACATCGAGTGCAACACAGACCTC  660

FcACS1 DQ269492.1   661   ATCCACATTGTCTACAGCCTCTCAGAGGACATGGGATTCCCTGGCTTCAGAGTCGGCGTT  720
FcACS1L             661   ATCCACATTGTTTACAGCCTCTCAAAGGACATGGGATTCCCTGGCTTCAGAGTCGGCGTT  720

FcACS1 DQ269492.1   721   GTCTACTCCTACAACGACGCGGTGGTGAATTGCGCGCGAAAGATGTCAAGCTTCGGATTA  780
FcACS1L             721   GTCTACTCCTACAACGACGCGGTGGTGAATTGCGCGCGAAAGATGTCTAGCTTCGGATTA  780

FcACS1 DQ269492.1   781   GTCTCGTCTCAAACACAACACCTGATTGCAGCCATGTTGTCGGATGATGTGTTTGTGACG  840
FcACS1L             781   GTCTCGTCTCAAACACAACACCTGATTGCAGCCATGTTGTCGGATGATGTGTTTGTGACG  840

FcACS1 DQ269492.1   841   AGGTTTTTGGCCGAGAGCGCTAGAAGATTGGAAAGAAGGCACAACGGCTTCACCCGAGGA  900
FcACS1L             841   AGGTTTTTGGCCGAGAGCGCTAGAAGATTGGAAAGAAGGCACAACGGCTTCACCCGAGGA  900

FcACS1 DQ269492.1   901   CTCGCTCAGGTTAGAATTACCTGCTTGGAGAGCAATGCAGGCCTGTTTTTGTGGATGGAT  960
FcACS1L             901   CTCGCTCAGGTTAGAATTACCTGCTTGGAGAGCAATGCAGGCCTGTTTTTGTGGATGGAT  960

FcACS1 DQ269492.1   961   TTGCACAGGCTTCTCAAAGAGCAGACACTTGATGCAGAGATGGCTCTATGGAAAGTCATC  1020
FcACS1L             961   TTGCACAGGCTTCTCAAAGAACAGACACTTGATGCAGAGATGGCTCTATGGAAACTCATC  1020

FcACS1 DQ269492.1   1021  ATTAATGAAGTTAAGCTCAACGTTTCGCCCGG-TTTTCGTTTCATTGCCCCGAACCGGGC 1079
FcACS1L             1021  ATTAATGAAGTTAAGCTCAACGTTTCGCCCGGTTTTTCGTTTCATTGCCCCGAACCGGGT 1080

FcACS1 DQ269492.1   1080  TGGTTCAGCGTATG---------------------------------------------- 1093
FcACS1L             1081  TGGTTCAGGGTGTGCATTGCCAACATGGATGATCACACCATGGAAATAGCTTTGACAAGA 1140

FcACS1 DQ269492.1   1093  ------------------------------------------------------------ 1093
FcACS1L             1141  ATACGAAACTTTGTGCTCCAAAAGAAGGAGCTTGTTGTGAAGCCAAGTAAGAAGAAGTGC 1200

FcACS1 DQ269492.1   1093  ------------------------------------------------------------ 1093
FcACS1L             1201  TGGCAAGATCACAACCTCAGGCTCAGCTTGAAAGCTCGCCGATTCGACGATTTCTTGATG 1260

FcACS1 DQ269492.1   1093  ----------------------GTTCGCCTGCAGTTTA------------- 1109
FcACS1L             1261  TCGCCTCATTCTCCTATTTCTCAATCACCTTTAGTCCAAGCAAGAACTTAA 1311
```

Fig. S2. Phylogenetic analysis of *FcACS* predicted proteins. The three types of ACS proteins are noted. Amino acid sequences of ACSs from tomato and apple were obtained from the NCBI database (http://www.ncbi.nlm.nih.gov/). NCBI accession numbers of the proteins are as follows: SlACS7 (AAK72432.1), SlACS8 (NP_001234160.1), SlACS3 (NP_001234026.1), MdACS1 (ABA03056.1), SlACS5 (NP_001234156.1), MdACS3a (AAB67989.1), MdACS3b (BAE94691.1), MdACS3c (BAE94692.1), SlACS2 (NP_001234178.1), SlACS4 (NP_001234280.1), MdACS5a (NP_001280768.1), MdACS5b (NP_001280870.1), SlACS1a (AAF97614.1), SlACS1b (AAF97615.1), SlACS6 (AAK72433.1) and PgACS1 (ABM60747.1)*. Alignment was performed by MUSCLE program using default parameters (http://www.ebi.ac.uk/Tools/msa/muscle) (Edgar, 2004). A phylogenetic tree was reconstructed based on the maximum likelihood (ML) framework using PhyML software (Guindon and Gascuel, 2003) based on the JTT matrix-based model (Jones *et al.*, 1992). A bootstrap consensus tree was inferred from 1,000 replicates and the number of replicate trees in which the associated clade clustered together in the bootstrap test is designated next to the branches (Felsenstein, 1985). The tree was graphically designed with the use of Fig Tree version 1.4 (http://tree.bio.ed.ac.uk/software/figtree/). *PgACS1 (*Picea glauca* L. ACS1) was defined as an outgroup.

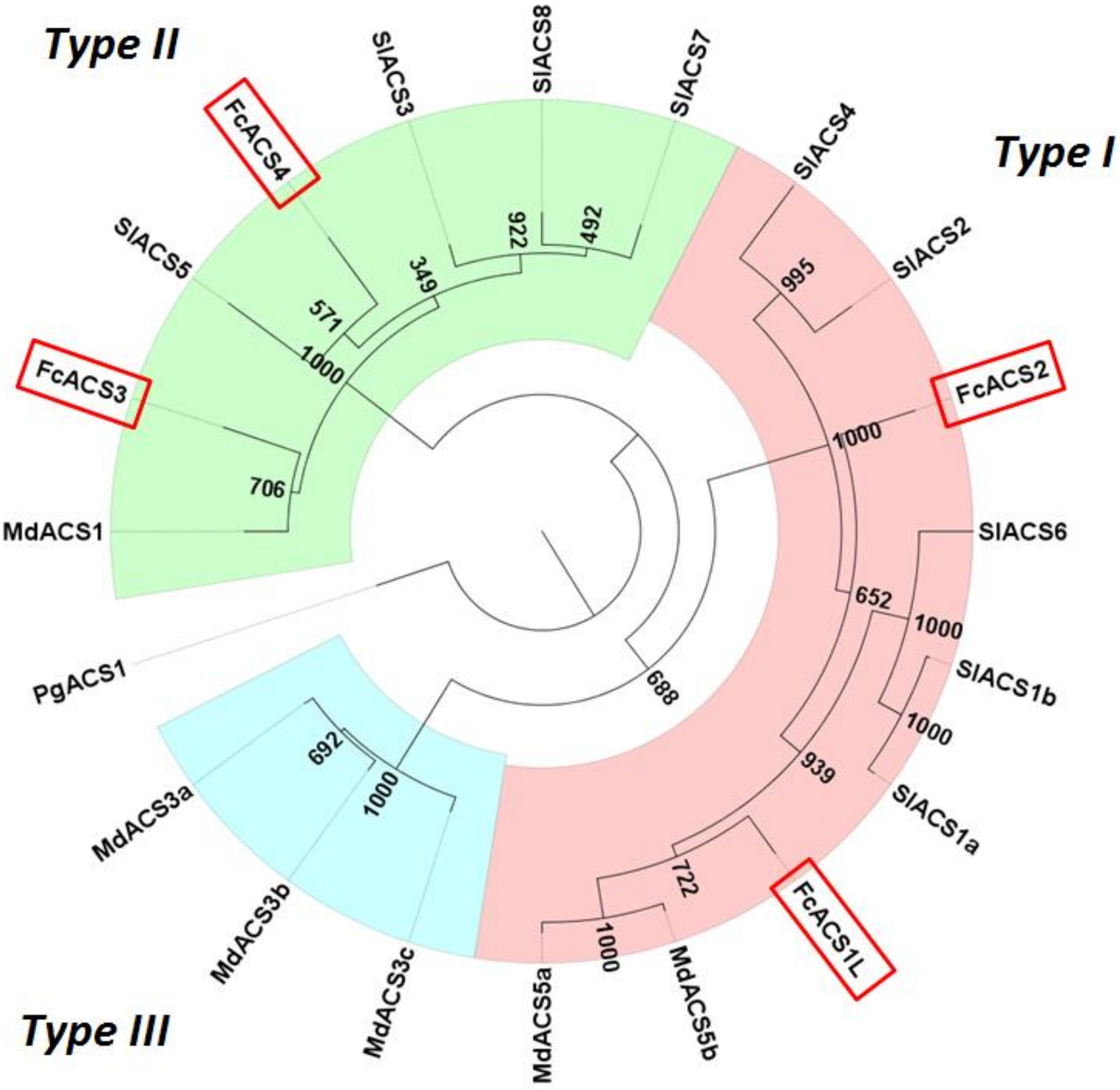

Type I
Type II
Type III
SlACS1a
SlACS1b
SlACS2
SlACS3
SlACS4
SlACS5
SlACS6
SlACS7
SlACS8
FcACS1L
FcACS2
FcACS3
FcACS4
MdACS1
MdACS3a
MdACS3b
MdACS3c
MdACS5a
MdACS5b
PgACS1
995
1000
652
1000
1000
939
722
1000
688
1000
692
706
1000
571
349
922
492

Fig. S3. Isolated *FcACOL* transcript (published previously under NCBI accession number AB307720.1 2007) aligned with the published sequence of *FcACO1* (Owino *et al*., 2006). Alignment was performed by MUSCLE program using default parameters (http://www.ebi.ac.uk/Tools/msa/muscle) (Edgar, 2004).

```
FcACOL              1   ATGGAGCAGATCACTTTTCCAGTTATTGACATGACAAAACTCAACGGCGACGAGAGAAAG 60
FcACO1|DQ269495.1   1   ----CGCGGATC------------------------------------------------ 8

FcACOL              61  ACAACCATGGAATTGATAAACGATGCTTGTGAG-AATTGGGGCTTCTTTGAGGTGATGAA 119
FcACO1|DQ269495.1   8   -----------------------CGCTTGTCAGAAATGGGGGGTTCTTTGAGGTGATGAA 45

FcACOL              120 CCATGGGATATCCCACGAGCTCATGGACACGGTGCAGAGGCTGACAAAGGAGCACTACAG 179
FcACO1|DQ269495.1   46  CCATGGGATATCCCACGAGCTCATGGACACGGTGCAGAGGCTGACAAAGGAGCACTACAG 105

FcACOL              180 AAAGTGCATGGAACAAAGGTTCAAGGAAATGGTGGCAACCAAGGGACTTGATGTTGTTCA 239
FcACO1|DQ269495.1   106 AAAGTGCATGGAACAAAGGTTCAAGGAAATGGTGGCAACCAAGGGACTTGATGTTGTTCA 165

FcACOL              240 GTCTGAAATCAAGGACTTGGACTGGGAAAGCACCTTCTTCTTGCGCCACCTTCCCGTCTC 299
FcACO1|DQ269495.1   166 GTCTGAAATCAAGGACTTGGACTGGGAAAGCACCTTCTTCTTGCGCCACCTTCCCGTCTC 225

FcACOL              300 AAACATGTCCGAAATCCCCGATCTCGGCGACGACTACAGGAAGGTAATGAAGGAATTTGC 359
FcACO1|DQ269495.1   226 AAACATGTCCGAGATCCCCGATCTCGGCGACGACTACAGGAAGGTAATGAAGGAATTTGC 285

FcACOL              360 TGCGGAGCTGGAGAAACTGGCAGAGCATCTCTTGGACTTGCTGTGTGAGAATCTTGGACT 419
FcACO1|DQ269495.1   286 TGCGGACTGGGAGAAACTGGCAGAGCATCTCTTGGACTTGCTGTGTGAGAATCTTGGACT 345

FcACOL              420 AGAGAAAGGGTACATAAAGAGCGTGTTCTATGGGTCAAAGGGTCCAAATTTTGGAACCAA 479
FcACO1|DQ269495.1   346 AGAGAGAGGGTACATAAAGAGCGTGTTCTATGGGTCAAAGGGTCCAAATTTTGGAACCAA 405

FcACOL              480 GGTGAGTAACTACCCTCCATGCCCCAAACCAGACCTGATCAAGGGCCTCCGGGCCCACAC 539
FcACO1|DQ269495.1   406 GGTGAGTAACTACCCTCCATGCCCCAAACCAGACCTGATCAAGGGCCTCCGGGCCAACAC 465

FcACOL              540 CGATGCCGGCGGCATCATCTTGCTCTTCCAAGACGACAAGGTCAGCGGTCTTCAGCTCCT 599
FcACO1|DQ269495.1   466 CGATGCCGGCGGTCATTTCTTGCTCTTCCAAGACGACAAGGTCAACGGTCTTCAGCTGCT 525

FcACOL              600 CAAGGATGACCAGTGGATTGATGTCCCTCCAATGCGCCACTCCATCGTCATCAACTTGGG 659
FcACO1|DQ269495.1   526 CAAGGATGACCAGTGGATTGATGTCCCTCCAGTGCGCCACTCCATCGTCATCAACTTGGG 585

FcACOL              660 TGACCAACTTGAGGTAATCACC-AATGGGCGATACAAGAGCGTGATGCACCGTGTGGTAG 718
FcACO1|DQ269495.1   586 TGAACAACTTGAGGTAATCACCAAATGGGCGATACAAGAGCGTGATGCACCG-GTGGTAG 644

FcACOL              719 CTCAACCGGACGGTAACAGGATGTCCCTAGCATCGTTCTACAACCCGGGAAGCGACGCGG 778
FcACO1|DQ269495.1   645 CTCAACCGGACGGTAACAGGATTTCCCTAGCATCGGTCTACCACCCGGGGAGCGACGGGG 704

FcACOL              779 TGATCTACCCAGCTCCGGCA-TTGGTGGAGAAAGCGGCGGAGCAGGAAACCAAAGTGGTG 837
FcACO1|DQ269495.1   705 TGATCTACCCAGCTCCGGCATTTGGTGGAAAAAGCCGCGGACAGGAAAACCAAAGTGGTG 764

FcACOL              838 TACCCAAAGTTCGTGTTTGATGACTACATGAAGCTGTATGCCGGGTTGAAATTCCAGGCA 897
FcACO1|DQ269495.1   765 TACCCAAA-TTCGTGTTTGATGACTACATGAGGCTGTATGCCCGGTTGAAATTTCCAGCC 823

FcACOL              898 AAGGAGCCTAGGTTTGAAGCCATGAAGGCCGTGGAATCCCCACTCAACGTGGATCCCATT 957
FcACO1|DQ269495.1   824 AAAGAGCC----------------------------CCTTCATTTAAT-CAAATTC---- 850

FcACOL              958 GCAACTGTTTGA 969
FcACO1|DQ269495.1   850 ------------ 850
```

Fig. S4. Alignment of deduced FcMADS8 amino acid sequence and SlRIN protein (NCBI accession number ABJ98752.1). Marked above sequence: red line – MADS-box domain; blue line – K-box domain; green line – C terminus. Black highlighted amino acids – identical residues; gray highlighted – similar residues. Alignment was performed by MUSCLE program using default parameters (http://www.ebi.ac.uk/Tools/msa/muscle) (Edgar, 2004).

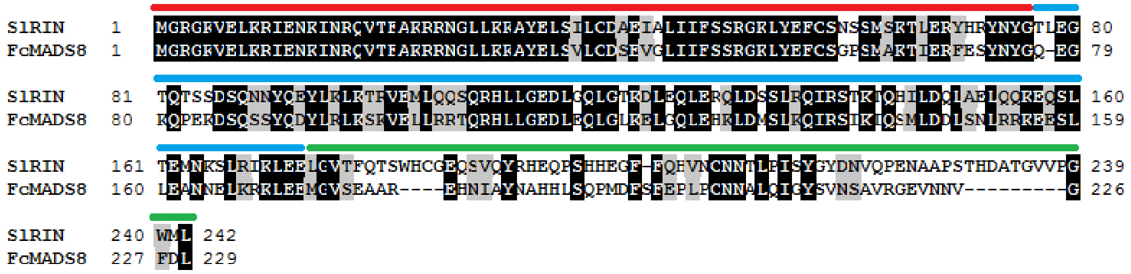

References

Edgar RC. 2004. MUSCLE: multiple sequence alignment with high accuracy and high throughput. *Nucleic Acids Research* **32**, 1792–1797.

Felsenstein J. 1985. Confidence limits on phylogenies: an approach using the bootstrap. *Evolution* **39**, 783–791.

Guindon S, Gascuel O. 2003. A simple, fast, and accurate algorithm to estimate large phylogenies by maximum likelihood. *Systematic Biology* **52**, 696–704.

Jones DT, Taylor WR, Thornton JM. 1992. The rapid generation of mutation data matrices from protein sequences. *Computer Application in the Biosciences* **8**, 275–282.

Owino WO, Manabe Y, Mathooko FM, Kubo Y, Inaba A. 2006. Regulatory mechanisms of ethylene biosynthesis in response to various stimuli during maturation and ripening in fig fruit (*Ficus carica* L.). *Plant Physiology and Biochemistry* **44**, 335–342.
